# Genome Analysis Linking Recent European and African Influenza (H5N1) Viruses

**DOI:** 10.3201/eid1305.070013

**Published:** 2007-05

**Authors:** Steven L. Salzberg, Carl Kingsford, Giovanni Cattoli, David J. Spiro, Daniel A. Janies, Mona Mehrez Aly, Ian H. Brown, Emmanuel Couacy-Hymann, Gian Mario De Mia, Do Huu Dung, Annalisa Guercio, Tony Joannis, Ali Safar Maken Ali, Azizullah Osmani, Iolanda Padalino, Magdi D. Saad, Vladimir Savić, Naomi A. Sengamalay, Samuel Yingst, Jennifer Zaborsky, Olga Zorman-Rojs, Elodie Ghedin, Ilaria Capua

**Affiliations:** * University of Maryland Center for Bioinformatics and Computational Biology, College Park, Maryland, USA; † Istituto Zooprofilattico Sperimentale delle Venezie, Padova, Italy; ‡ The Institute for Genomic Research, Rockville, Maryland, USA; § Ohio State University, Columbus, Ohio, USA; ¶ Animal Health Research Institute, Giza, Egypt; # Veterinary Laboratories Agency, Addlestone, England, UK; ** Central Laboratory of Animal Pathology, Bingerville, Côte d’Ivoire; †† Istituto Zooprofilattico Sperimentale dell’Umbria e delle Marche, Perugia, Italy; ‡‡ Department of Animal Health, Hanoi, Vietnam; §§ Istituto Zooprofilattico Sperimentale della Sicilia, Palermo, Italy; ¶¶ National Veterinary Research Institute, Vom. Plateau State, Nigeria; ## Food and Agriculture Office of the United Nations, Tehran, Iran; *** Ministry of Agriculture, Animal Husbandry and Food, Kabul, Afghanistan; ††† Istituto Zooprofilattico Sperimentale della Puglia e Basilicata, Foggia, Italy; ‡‡‡ US Naval Medical Research Unit No. 3, Cairo, Egypt; §§§ Croatian Veterinary Institute, Zagreb, Croatia; ¶¶¶ University of Ljubljana, Ljubljana, Slovenia; ### University of Pittsburgh School of Medicine, Pittsburgh, Pennsylvania, USA

**Keywords:** Influenza A virus, genomics, sequence analysis, DNA, evolution, molecular, research

## Abstract

To better understand the ecology and epidemiology of the highly pathogenic avian influenza virus in its transcontinental spread, we sequenced and analyzed the complete genomes of 36 recent influenza A (H5N1) viruses collected from birds in Europe, northern Africa, and southeastern Asia. These sequences, among the first complete genomes of influenza (H5N1) viruses outside Asia, clearly depict the lineages now infecting wild and domestic birds in Europe and Africa and show the relationships among these isolates and other strains affecting both birds and humans. The isolates fall into 3 distinct lineages, 1 of which contains all known non-Asian isolates. This new Euro-African lineage, which was the cause of several recent (2006) fatal human infections in Egypt and Iraq, has been introduced at least 3 times into the European-African region and has split into 3 distinct, independently evolving sublineages. One isolate provides evidence that 2 of these sublineages have recently reassorted.

## Abstract 2

Although linked, these viruses are distinct from earlier outbreak strains.

The first cases of human infection with highly pathogenic avian influenza (HPAI) strain H5N1 occurred in Hong Kong in 1997; it was brought under control by massive culling of the chicken population (*1*,*2*). An antigenically distinct strain emerged in 2002, in the same location, and has since spread to hundreds of millions of birds (*3*,*4*). More alarming has been the growing number of human influenza (H5N1) infections; by September 2006, 251 human cases had been reported, resulting in 148 deaths (*2*). From late 2005 to early 2006, HPAI (H5N1) was detected for the first time in birds in eastern Europe, the Middle East, and northern Africa, indications that the virus was spreading, possibly aided by wild bird migration. Human cases were reported beginning in January 2006 in Egypt, Iraq, Turkey, Djibouti, and Azerbaijan.

## Methods

We sequenced and analyzed the genomes of 36 recent isolates of highly pathogenic influenza (H5N1) viruses collected from Europe, northern Africa, the Middle East, and Asia. We used high-throughput methods described previously (*5*).

### Sample Collection

Samples primarily consisting of pooled trachea and lung tissue, pooled intestines, or tracheal and cloacal swabs collected from dead or moribund animals were processed for attempted virus isolation as described (*6*). Hemagglutinating isolates were typed either by reverse transcription–PCR (RT-PCR) or by serologic methods (*7*). RNA was extracted with the High Pure Extraction Kit (Roche, Indianapolis, IN, USA), according to manufacturer’s instructions.

### Primer Design

Sequences from recent human and avian influenza (H5N1) isolates were downloaded from GenBank and were aligned with MUSCLE (*8*). Degenerate primers were designed on the basis of consensus sequences generated with BioEdit (*9*). An M13 sequence tag was added to the 5′ end of each primer to be used for sequencing. Four of the reactions were analyzed by electrophoresis on an agarose gel for quality control purposes. Primer design was optimized by analysis of the sequence success rate of each primer pair. Primers that did not perform well were redesigned and replaced in the primer set. Primers were designed to produce ≈500-nt overlapping amplicons to provide 2× coverage of each genomic segment. Additionally, a second set of primers was designed to produce 500-nt amplicons offset ≈250 nt from the original primer pair, which gave at least 4× sequence coverage of each segment.

### cDNA Synthesis

Amplicons tiling the genome of the influenza isolates were generated with a OneStep RT-PCR kit (QIAGEN, Valencia, CA, USA). They were treated with shrimp alkaline phosphatase-exonuclease I (U.S. Biologicals, Swampscott, MA, USA) before sequencing.

### Sequencing and Assembly

Sequencing reactions were performed as described previously (*5*). After sequencing, each segment was downloaded, trimmed to remove amplicon primer-linker sequence as well as low-quality sequence, and assembled. A small genome assembler called Elvira, based on the open-source Minimus assembler (http://cbcb.umd.edu/software), has been developed to automate these tasks. The Elvira pipeline delivers exceptions, including failed reads, failed amplicons, insufficient coverage of a reference sequence (as obtained from GenBank), ambiguous consensus sequence calls, and low-coverage areas. Additional sequencing and targeted RT-PCR were conducted to close gaps and to increase coverage in low-coverage or ambiguous regions.

All sequence data used in this study are available from GenBank and also from ftp.cbcb.umd.edu/pub/data/flu. GenBank accession numbers are available in the supplementary data (Technical Appendix 1).

### Phylogenetic Analysis

Multiple sequence alignments of nucleotide data were performed by using MUSCLE (*8*) with default parameters. Most alignments of segments within a subtype lack internal gaps. Leading and trailing gaps were not considered in tree-length calculations, but all nucleotide positions were considered.

**Figure 1:**
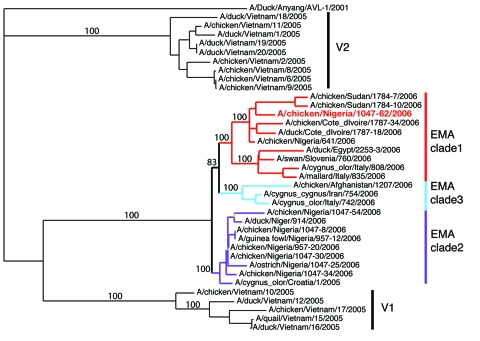
Phylogenetic tree of hemagglutinin (HA) segments from 36 avian influenza samples. A 2001 strain (A/duck/Anyang/AVL-1/2001) is used as an outgroup at top. Clade V1 comprises the 5 Vietnamese isolates at the bottom of the tree, and clade V2 comprises the 9 Vietnamese isolates near the top of the tree. The European-Middle Eastern-African (EMA) clade contains the remaining 22 isolates sequenced in this study; the 3 subclades are indicated by red, blue, and purple lines. The reassortant strain, A/chicken/Nigeria/1047–62/2006, is highlighted in red. Note that 4 segments including HA from this reassortant fall in EMA-1; the other 4 fall in EMA-2, as shown in Appendix Figure 1. Bootstrap values supporting the 3 distinct EMA clades are taken from a consensus tree based on concatenated whole-genome sequences, excluding the reassortant strain. The consensus tree is provided as Appendix Figure 2.

**Figure 2:**
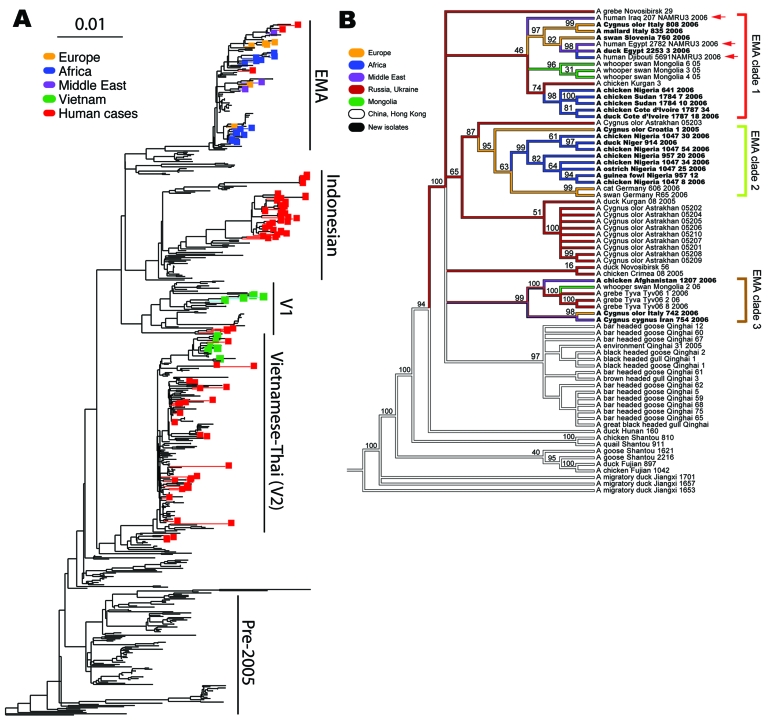
A) Phylogenetic tree relating the influenza A (H5N1) hemagglutinin (HA) segments of 589 avian, feline, and human viruses. The tree includes all HA segments isolated since 2000 from humans (82 isolates, minimum sequence length 1,000 nt), birds (503 isolates, minimum length 1500 nt), and cats (4 isolates). The 36 newly sequenced genomes are highlighted in color. Human cases, which occur in all 4 of the major influenza (H5N1) clades, are highlighted in red. The scale bar indicates an F84 distance of 0.01. A full-scale version of this tree is provided as Figure 3. B) Phylogeny of 71 complete genomes (avian isolates, all 8 segments concatenated) and 3 HA sequences (human isolates, marked with red arrows) from Europe, the Middle East, Africa, Russia, and Asia. Bootstrap values represent the percentage of 1,000 bootstrap replicates for which the partition implied by the edge was observed; see Methods for further details. The 3 European-Middle Eastern-African (EMA) subclades from Figure 1 are indicated with the same color scheme. Isolates from human hosts are found only in EMA-1. Colors indicate locales. The names of the isolates newly sequenced in this study are shown in **boldface** text.

The phylogenetic trees for Figures 1, 2A, and Appendix Figures 1–3 were constructed by using the neighbor-joining method as implemented in PAUP* version 4.0b10 (*10*,*11*) using the F84 distance between nucleotide sequences and the default parameters. The phylogeny of 71 complete genomes (avian isolates) and 3 hemagglutinin (HA) sequences (human isolates) in Figure 2B comprises isolates chosen because they formed the European-Middle Eastern-African (EMA) clades and the Russian and Chinese sister clades in a larger analysis of 759 influenza (H5N1) isolates from the locales and host range of all H5N1 sequences published since 1996. The figure includes every member of the EMA clade for which the complete genome sequence is currently available, except chicken/Nigeria/1047–62/2006 and chicken/Kurgan/05/2005, which appear to be reassortants.

To find optimal phylogenetic trees for Figure 2B, we used a combination of tree search algorithms available in the “new technology” heuristic strategies in the TNT (*12*) software package (available from www.zmuc.dk/public/phylogeny/TNT). These strategies include a successive combination of hill-climbing techniques (branch swapping) followed by simulated annealing (ratcheting), divide-and-conquer (sectorial searches), and genetic algorithms (tree fusion). Figure 2B depicts a strict consensus based on 286 minimal-length trees resulting from a parsimony search of 1,000 replicates in TNT under the command “xmult = lev5.” Each component tree had a tree length of 1,613 steps. Gaps were treated as a fifth state, and all edit costs were given equal weights under the parsimony criterion. The heuristic tree strategy was run until a stable strict consensus was achieved. This strict consensus is a conservative estimate of the phylogenetic relationship between the isolates, where an edge is included only if it was observed in all 286 optimal trees. Separately, RAxML (*13*) was run over the same data for maximum likelihood analyses under the general time-reversible (GTR) mixed model of nucleotide substitution. This likelihood analysis produced a tree with the same clade contents as the parsimony tree, preserving the 3 EMA clades. Branches were traced with colors to represent the locale of isolation of the virus.

## Results and Discussion

The 36 new isolates reported here greatly expand the amount of whole-genome sequence data available from recent avian influenza (H5N1) isolates. Before our project, GenBank contained only 5 other complete genomes from Europe for the 2004–2006 period, and it contained no whole genomes from the Middle East or northern Africa. Our analysis showed several new findings. First, all European, Middle Eastern, and African samples fall into a clade that is distinct from other contemporary Asian clades, all of which share common ancestry with the original 1997 Hong Kong strain. Phylogenetic trees built on each of the 8 segments show a consistent picture of 3 lineages, as illustrated by the HA tree shown in Figure 1. Two of the clades contain exclusively Vietnamese isolates; the smaller of these, with 5 isolates, we label V1; the larger clade, with 9 isolates, is V2. The remaining 22 isolates all fall into a third, clearly distinct clade, labeled EMA, which comprises samples from Europe, the Middle East, and Africa. Trees for the other 7 segments display a similar topology, with clades V1, V2, and EMA clearly separated in each case. Analyses of all available complete influenza (H5N1) genomes and of 589 HA sequences placed the EMA clade as distinct from the major clades circulating in People’s Republic of China, Indonesia, and Southeast Asia.

The influenza (H5N1) viruses isolated in Europe, the Middle East, and Africa show a close relationship, despite the fact that they were collected from a widely dispersed geographic region, including Côte d’Ivoire, Nigeria, Niger, Sudan, Egypt, Afghanistan, Iran, Slovenia, Croatia, and Italy. The shared lineage of the viruses suggests a single genetic source for introduction of influenza (H5N1) into western Europe and northern and western Africa; our analysis places this source most recently in either Russia or Qinghai Province in China (Figure 2B; Appendix Table. The broad dispersal of these isolates throughout these countries during a relatively short period, coupled with weak biosecurity standards in place in most rural areas, implicates human-related movement of live poultry and poultry commodities as the source of introduction of influenza (H5N1) into some of these countries. The virus’ presence in wild birds leaves open the alternative possibility that migratory birds may have been the primary source, with secondary spread possibly caused by human-related activities.

A phylogenetic tree containing 589 isolates from 2001 through 2006 (Figure 2A and Appendix Figure 3) shows the relationship of the 36 recent isolates from this study to previous isolates and shows the 3 major lineages of influenza (H5N1) that are now circulating in Asia plus the fourth lineage, EMA, that has spread west into Europe and Africa. Figure 2B depicts a consensus view of the parsimony-based analysis of 74 isolates of complete genomes from the EMA lineage. The EMA clade contains all known European, Middle Eastern, and North African cases (which began appearing in late 2005), as well as cases from China, Russia, and Mongolia in 2005 and 2006. Some of the EMA clade isolates appear in clusters of influenza (H5N1) infection that were reported in geese in Qinghai Province, China (*14*), and in mute swans in Astrakhan (*15*), both of which are possible sources of spread through migration.

The evolutionary relationships shown in Figure 2B provide clear evidence that 3 distinct clades, labeled EMA 1–3, are circulating in the European and African region. These clades clearly share a common ancestor in Asia. The 3 clades may represent separate introductions or, alternatively, a single introduction from Asia into Russia, Europe, or another western site that has subsequently evolved into 3 lineages. More data will be required to pinpoint when and where the 3 clades split apart. All previously reported European and Middle Eastern isolates belong to EMA-1.

Our results show that EMA-2 has spread to Europe and that EMA-3 has spread to both Europe and the Middle East. These results agree in part with a recent study (*16*) that reported 3 distinct introductions of influenza (H5N1) into Nigeria. Our analysis, based on all available HA sequences (Appendix Figure 3), indicates that the Nigerian isolates fall into just 2 clades, EMA 1–2, that likely resulted from at least 2 introductions of influenza (H5N1).

European countries have been affected by each of the 3 introductions of the EMA strains. For example, the Italian sequences can be segregated into 2 subgroups (Figure 2B). Two isolates in EMA-1 (Co/Italy/808/06 and Md/Italy/835/2006) are closely related in all segments and likely share a common ancestor with isolates found in Slovenia (Sw/Slovenia/760/2006), Bavaria, and the Czech Republic (Co/Czech Republic/5170/2006). The third Italian strain from our study (Co/Italy/742/2006) falls into EMA-3, along with our newly sequenced isolates from Iran (Co/Iran/754/2006) and Afghanistan (Ck/Afghanistan/1207/2006). EMA-2 contains 1 European isolate, from a swan in Croatia, and multiple isolates from domesticated birds in Nigeria and Niger. This group shares a common ancestor with a group of isolates from Astrakhan and Kurgan (Russia).

Of the 22 EMA isolates newly sequenced in this study, 20 have the amino acid lysine (K) at position 627 of the polymerase basic protein 2 (PB2), while only 2 have glutamic acid (E). (These last 2 are both from Italy and both in EMA-1.) The 627K mutation is associated with virulence in mice and adaptation to mammalian hosts (*17*) and with increased host range (*18*). Lysine at this position is common in human viruses: all 65 human influenza (H5N1) isolates from 2001 through 2006 for which the PB2 sequence is available have lysine at position 627. Before the analysis of our collection, the PB2 627K was a relatively rare finding in avian influenza (H5N1) viruses: it was present in only 42 of 385 isolates previously collected from 2001 through 2006. Our analysis shows that all 42 of these fall in the EMA clade (Figure 2 and supplementary data available in Technical Appendix 2. Excluding our current European, Middle Eastern, and African isolates, this mutation appears primarily in isolates obtained from wild birds in Astrakhan (*15*) and at Qinghai Lake (*14*,*17*). This mutation also occurs in the recent isolate A/Guinea fowl/Shantou/1341/2006 and in a mouse-adapted 2001 Asian isolate, A/pheasant/Hong Kong/Fy155/01-MB. This finding is in keeping with current knowledge of the acquisition of such mutations.

Our study increases current knowledge on strains circulating in Asia before the westward spread of influenza A (H5N1). The Vietnamese samples fall into 2 clusters, the larger of which (V2 in Figure 1) is the same strain responsible for multiple cases in Southeast Asia since 2004, particularly in Vietnam and Thailand. These isolates all seem to derive from earlier Hong Kong samples (including 2 cases of human infection) in 2002 and 2003. The second cluster, V1, which contains 5 samples, significantly expands our understanding of this distinct Vietnamese influenza (H5N1) lineage. The only other isolate from this cluster was recently reported in a Vietnamese duck (A/duck/Vietnam/568/2005) and labeled a “recent Vietnam introduction” (*4*). This sample groups with the V1 clade when shown in the context of a larger tree of HA sequences (Appendix Figure 3). The 5 newly sequenced isolates in clade V1 show the same phylogenetic relationship for all segments except PB2 (Appendix Figure 1). The isolates in clade V1 appear to have undergone the same reassortment as was suggested (*4*) for the 1 previous example of this Vietnamese clade, A/duck/Vietnam/568/2005; i.e., they have acquired a new PB2 segment. This PB2 is similar to older (1996–2002), A/duck**/**Guangdong/1/96-like viruses from China. V1 clade isolates are associated with a distinct set of human cases, from China’s Anhui and Guangxi Provinces in 2005, a finding that provides additional support to the hypothesis that this group of influenza (H5N1) viruses was introduced into Vietnam from China (*4*).

Although EMA has split into 3 independently evolving clades, 1 isolate, A/chicken/Nigeria/1047–62/2006, shows clear evidence of reassortment. In this genome, 4 segments—HA, (nucleocapsid protein, nonstructural protein, and PB1—belong to EMA-1, as seen in Figure 1 and Appendix Figure 1. The other 4 segments—neuraminidase, matrix protein, PA, and PB2—belong to EMA-2 (Appendix Figure 1). Individual segment trees based on all available sequences in GenBank corroborate this pattern and consistently split the 8 segments of this Nigerian isolate into 2 distinct clades. Reassortment events such as this can only be discovered by sequencing multiple virus segments.

The presence of all 3 EMA sublineages in the same geographic region creates ample opportunities for reassortment. Isolate A/chicken/Nigeria/1047–62/2006 is the most recent of the Nigerian isolates, consistent with the hypothesis that this reassortant was generated in Africa. Additional surveillance will be necessary to determine if this reassortant strain spreads further in the avian population and to assess its ability to infect mammals.

As shown in Figure 2A, the EMA clade is a distinct lineage evolving independently of the 3 exclusively Asian lineages. All 3 human influenza (H5N1) cases that have been sequenced outside east Asia—from Iraq (*19*), Djibouti, and Egypt—belong to the EMA lineage. The human sequences A/Djibouti/5691/NAMRU3/06 and A/Egypt/2782/NAMRU3/06 group closely together and consistently fall in EMA-1. The placement of A/Iraq/207/NAMRU3/06 is slightly less certain; it also groups with EMA-1 (Figure 2B) but with lower bootstrap support. EMA viruses isolated from humans are thus quite distinct from the recent large clusters of human cases in Indonesia and China, which fall into separate clades containing none of our samples. The EMA isolates are also distinct from other human cases in Southeast Asia, which fall into the clades (V1 and V2) containing our Vietnamese samples.

The emergence of 3 (or more) substrains from the EMA clade represents multiple new opportunities for avian influenza (H5N1) to evolve into a human pandemic strain. In contrast to strains circulating in Southeast Asia, EMA viruses are derived from a progenitor that has the PB2 627K mutation. These viruses are expected to have enhanced replication characteristics in mammals, and indeed the spread of EMA has coincided with the rapid appearance of cases in mammals—including humans in Turkey, Egypt, Iraq, and Djibouti, and cats in Germany, Austria, and Iraq. Unfortunately, the EMA-type viruses appear to be as virulent as the exclusively Asian strains: of 34 human infections outside of Asia through mid-2006, 15 have been fatal (*2*).

Analyses of the complete HA tree (Figure 2A and Appendix Figure 3) suggest that the earliest sequenced relatives of the EMA clade are from the Yunnan region of China (A/duck/Yunnan/6255,6445/2003), Hong Kong, (A/chicken/Hong Kong/WF157/2003), and South Korea (A/chicken/Korea/ES/2003, A/duck/Korea/ESD1/2003), which were part of a regional outbreak in 2003 (*20*). Experiments on the 2 Korean isolates showed them to be infectious but not fatal in mice (*21*).

These findings show how whole-genome analysis of influenza (H5N1) viruses is instrumental to the better understanding of the evolution and epidemiology of this infection, which is now present in the 3 continents that contain most of the world’s population. This and related analyses, facilitated by global initiatives on sharing influenza data (*22*,*23*), will help us understand the dynamics of infection between wild and domesticated bird populations, which in turn should promote the development of control and prevention strategies.

## Supplementary Material

Appendix Figure 1 Phylogenetic trees of neuraminidase (NA), matrix protein (MP), nucleocapsid protein (NP), nonstructural protein (NS), polymerase acidic protein (PA), polymerase basic protein 1 (PB1), and polymerase basic protein 2 (PB2) segments from the 36 influenza strains sequenced in this study. A 2001 strain is used as an outgroup. Tree constructed with PAUP (Swofford DL. PAUP*: Phylogenetic Analysis Using Parsimony [and Other Methods]. 4.0 Beta. Sunderland [MA]: Sinauer Associates; 2002) as explained in the Methods.

Appendix Figure 2 Consensus of 1,000 neighbor-joining bootstrap replicates as calculated by PAUP 4.0b10 (Swofford DL. PAUP*: Phylogenetic Analysis Using Parsimony [and Other Methods]. Sunderland [MA]: Sinauer Associates; 2002) on 35 of our new 36 isolates, leaving aside the reassortant strain A/Ck/Nigeria/1047 62/2006, which-because of its multilineage ancestry-cannot be placed exclusively in either EMA clade 1 or 2. Each isolate was represented by the concatenation of the alignments of its 8 RNA segments. These bootstrap values are used in the labeling of Figure 1 in the main text.

Appendix Figure 3 Phylogenetic tree relating hemagglutinin (HA) segments of 589 avian, feline, and human influenza A (H5N1) viruses. This figure is an enlarged view of Figure 2A in the main text. The tree includes all human-derived HA segments deposited in GenBank since the year 2000 that are at least 1000 nt in length (82 sequences) as well as all avian and feline sequences since the year 2000 that are at least 1500 nt in length (4 feline and 503 avian sequences). The sequences were aligned with MUSCLE (Edgar RC. MUSCLE: a multiple sequence alignment method with reduced time and space complexity. BMC Bioinformatics. 2004;5:113), and the tree was created with the neighbor joining algorithm of PAUP* version 4.0b10 (Swofford DL. PAUP*: Phylogenetic Analysis Using Parsimony [and Other Methods]. Sunderland [MA]: Sinauer Associates; 2002) using the F84 distance between the nucleotide sequences and the default parameters. The scale bar in the upper left indicates an F84 distance of 0.01. Human isolates in the figure are red. The V3 clade is pink. The 36 newly sequenced avian isolates are colored according to the region from which they were obtained (blue, Africa; orange, Europe; purple, Middle East; and greenish blue, Vietnam). The isolate A/chicken/Nigeria/1047_62/2006 that has undergone reassortment is marked with an arrow.

Appendix Table Isolate name

Technical Appendix 1 POSITION 627 IN PB2 PROTEIN FOR ALL 420 SEQUENCES AVAILABLE FROM 2001-2006 AS OF November 2006

Technical Appendix 2 1: CY017194 Influenza A virus (A/duck/Viet Nam/19/2005(H5N1)
