# Supplementary figures and images for: Genome Analysis Linking Recent European and African Influenza (H5N1) Viruses
Source: Emerg Infect Dis. 2007 May;13(5):713–8. doi: 10.3201/eid1305.070013 (PMC2432181; doi:10.3201/eid1305.070013)

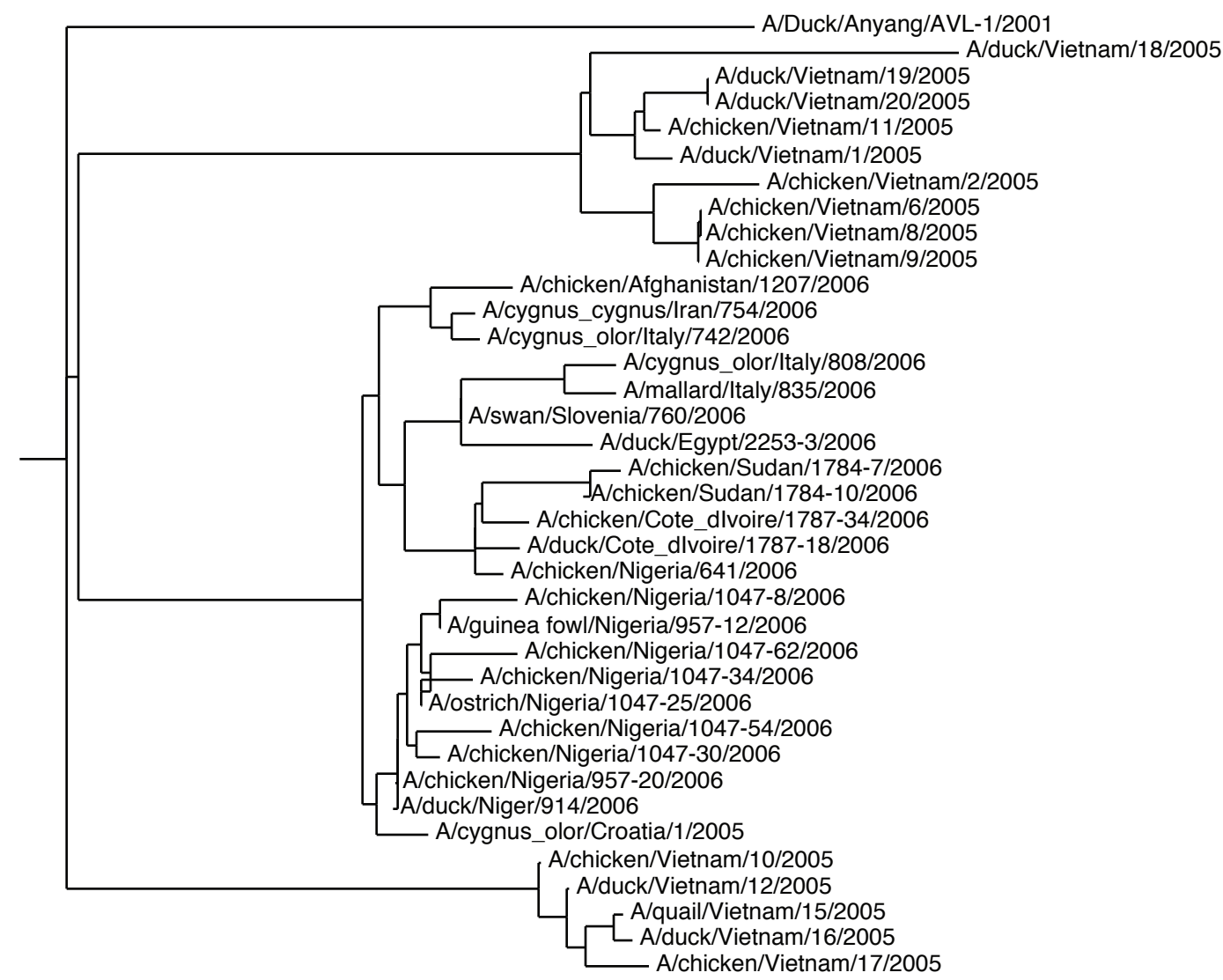

NA

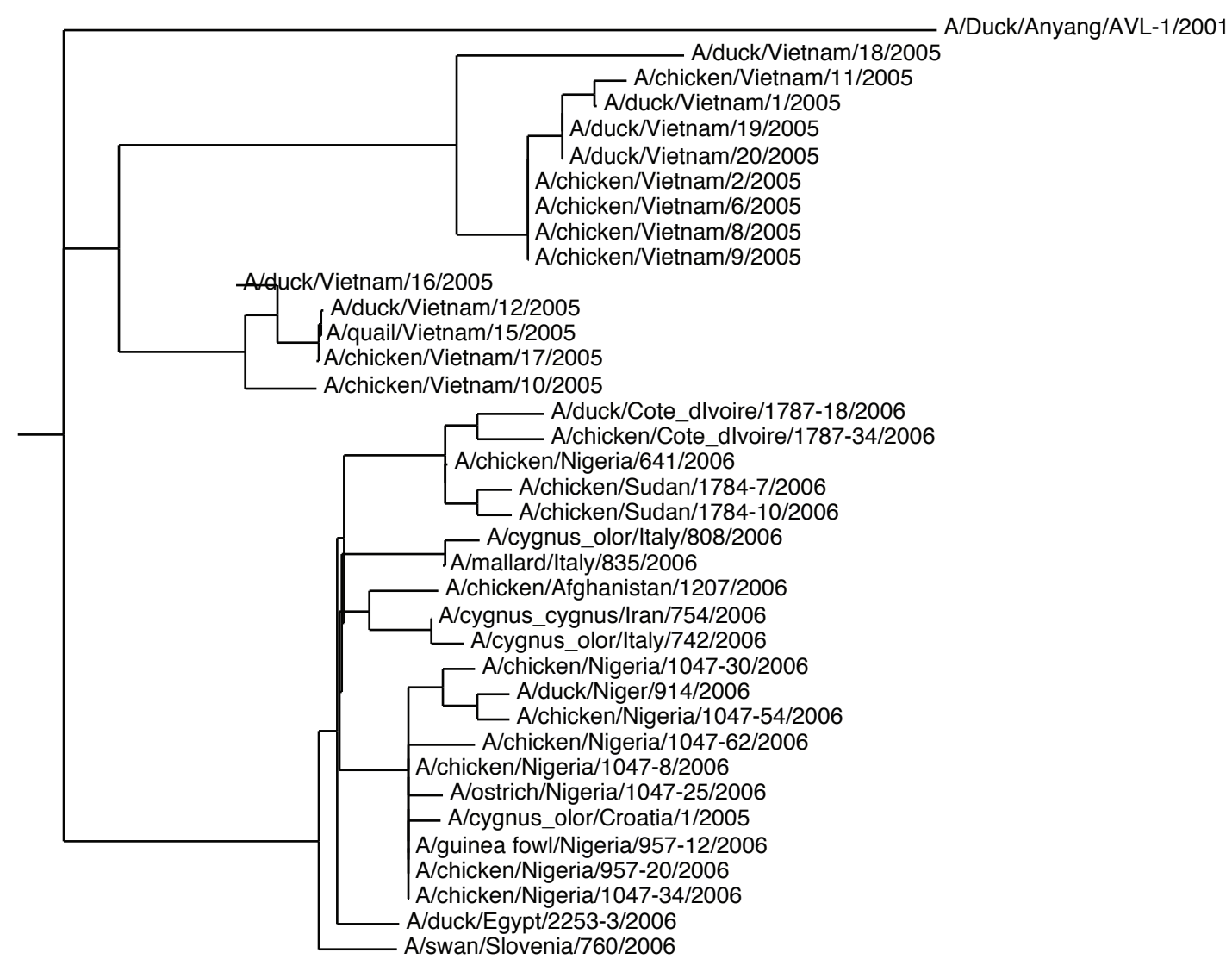

MP

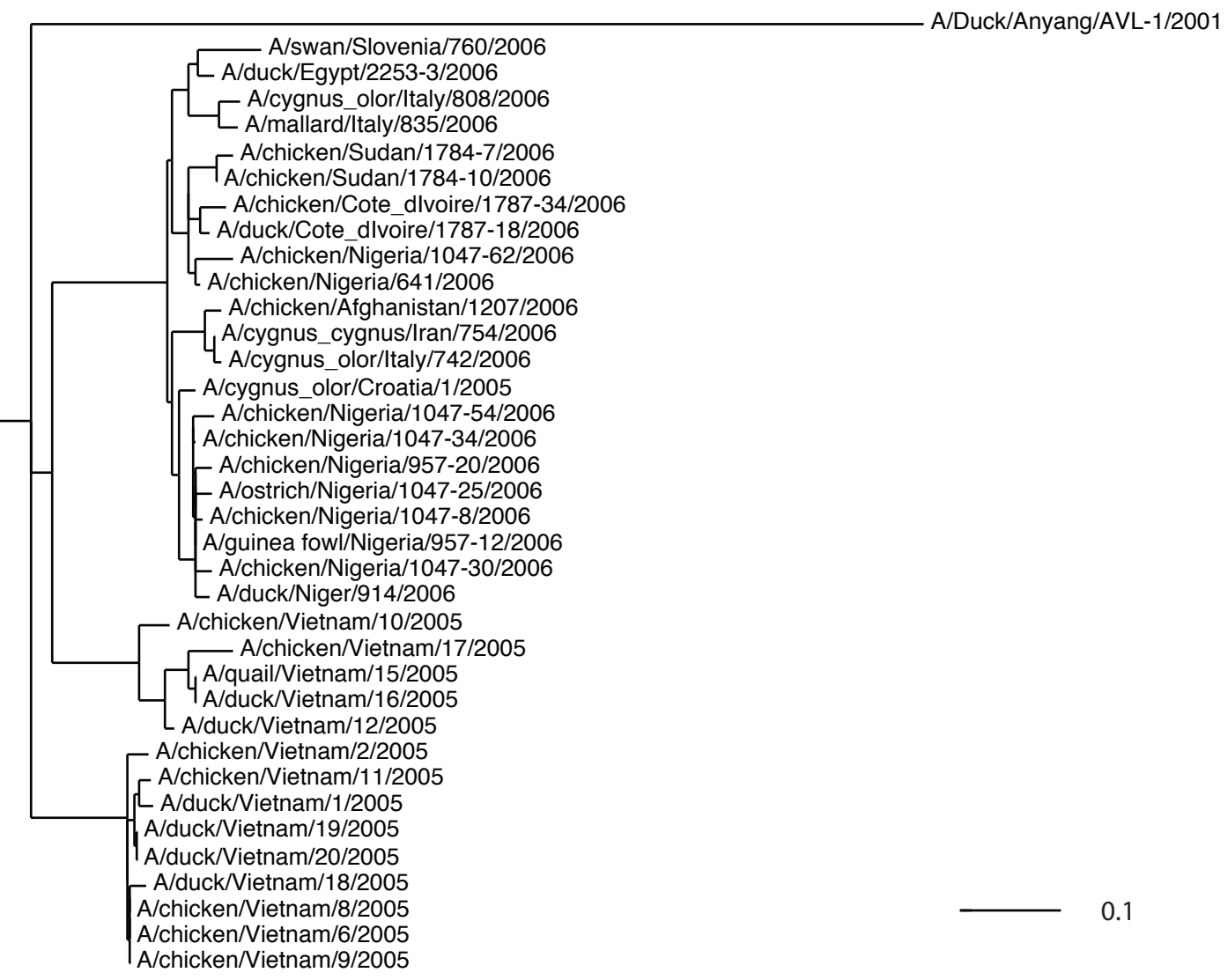

NP

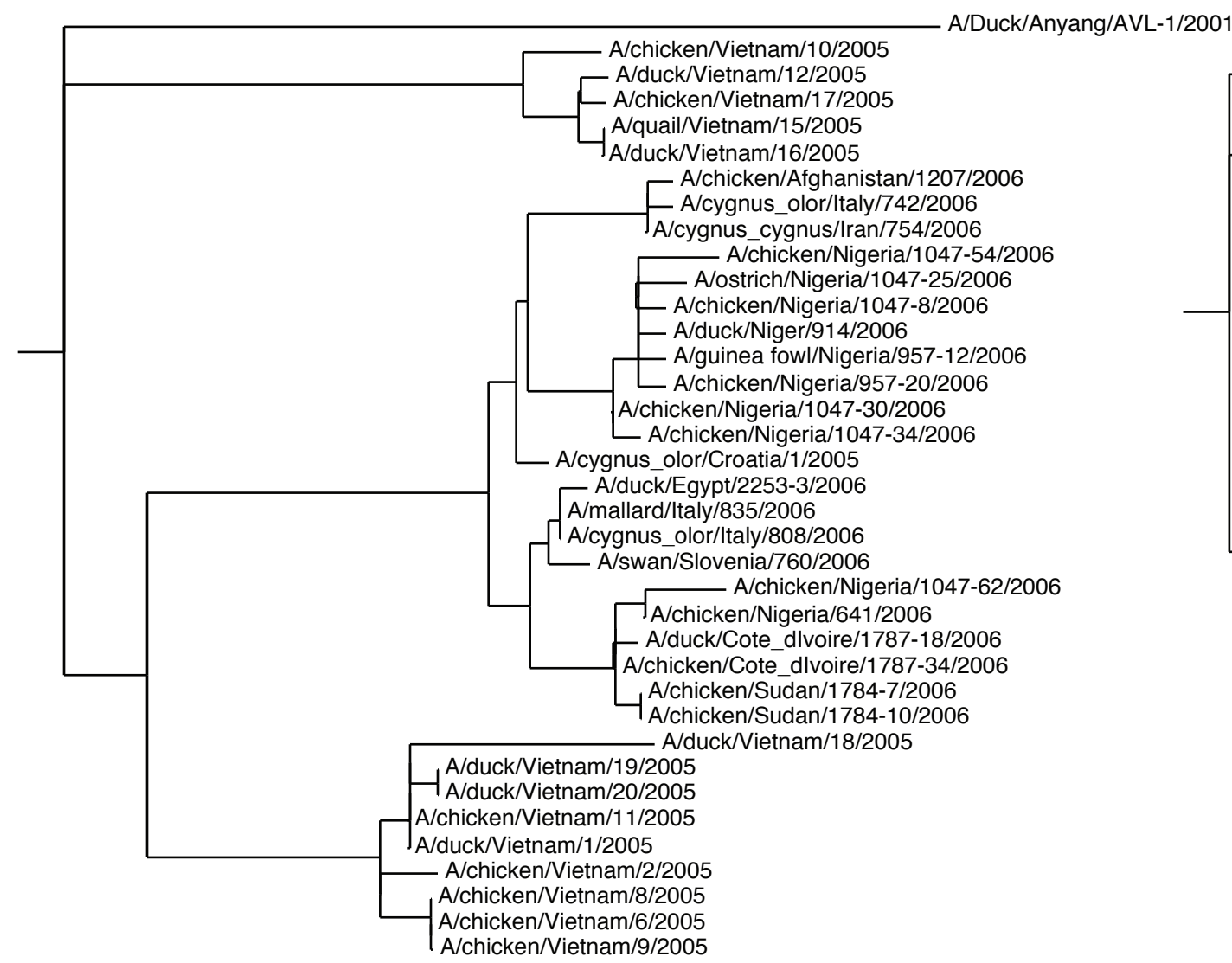

NS

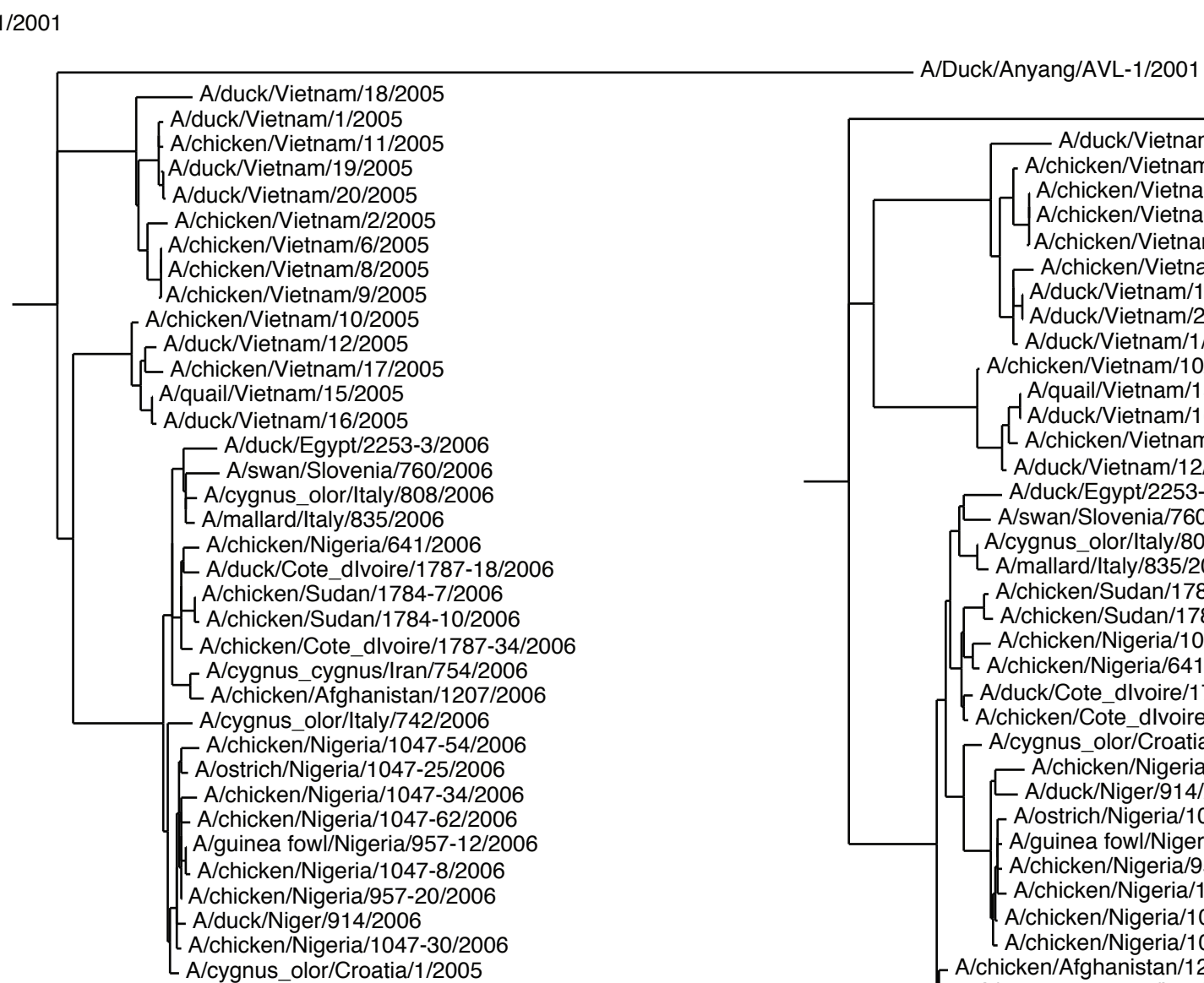

PA

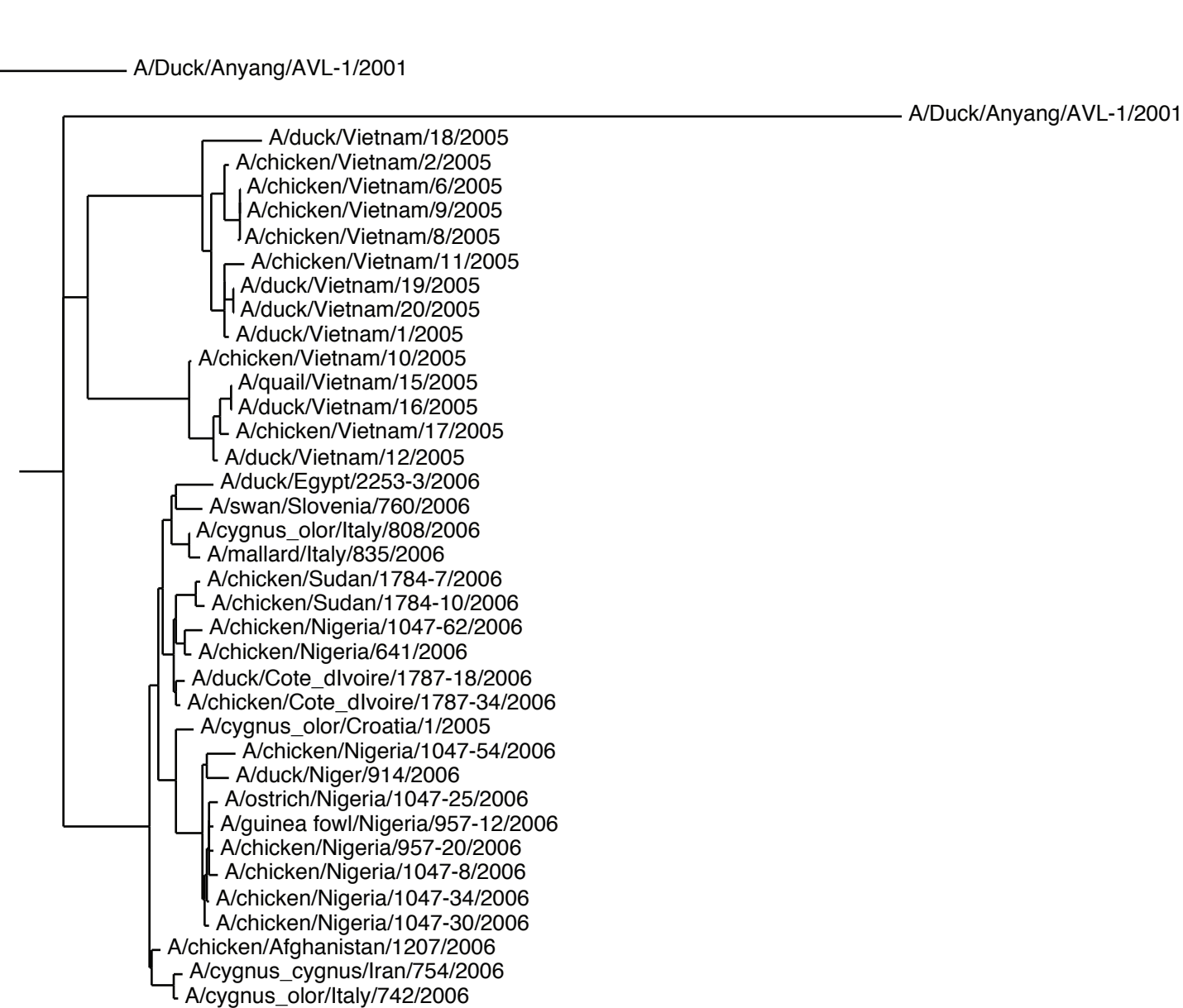

PB1

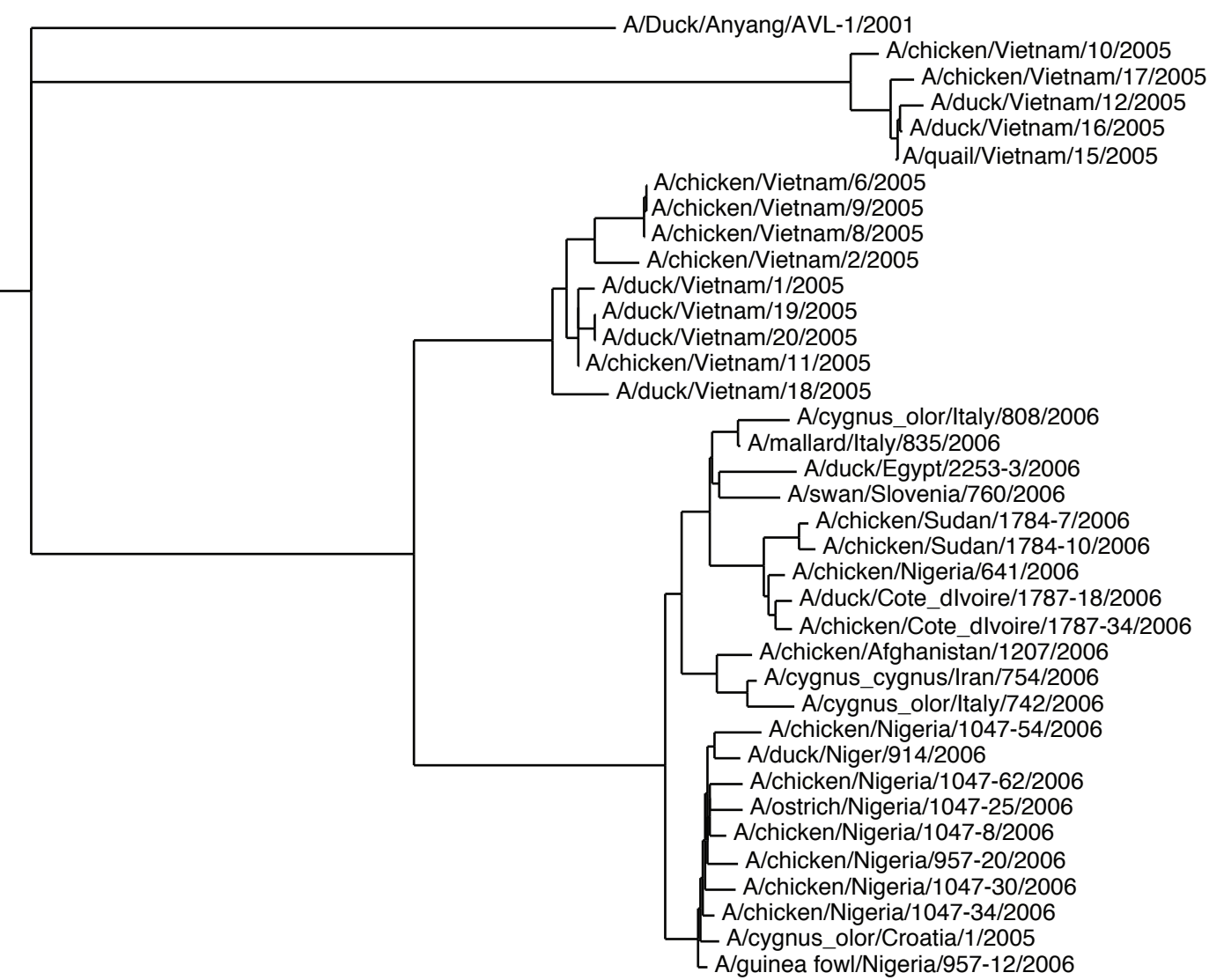

PB2

Supplement: Appendix Figure 1 — Phylogenetic trees of neuraminidase (NA), matrix protein (MP), nucleocapsid protein (NP), nonstructural protein (NS), polymerase acidic protein (PA), polymerase basic protein 1 (PB1), and polymerase basic protein 2 (PB2) segments from the 36 influenza strains sequenced in this study. A 2001 strain is used as an outgroup. Tree constructed with PAUP (Swofford DL. PAUP*: Phylogenetic Analysis Using Parsimony [and Other Methods]. 4.0 Beta. Sunderland [MA]: Sinauer Associates; 2002) as explained in the Methods. [file 07-0013_appF1-s1.pdf]

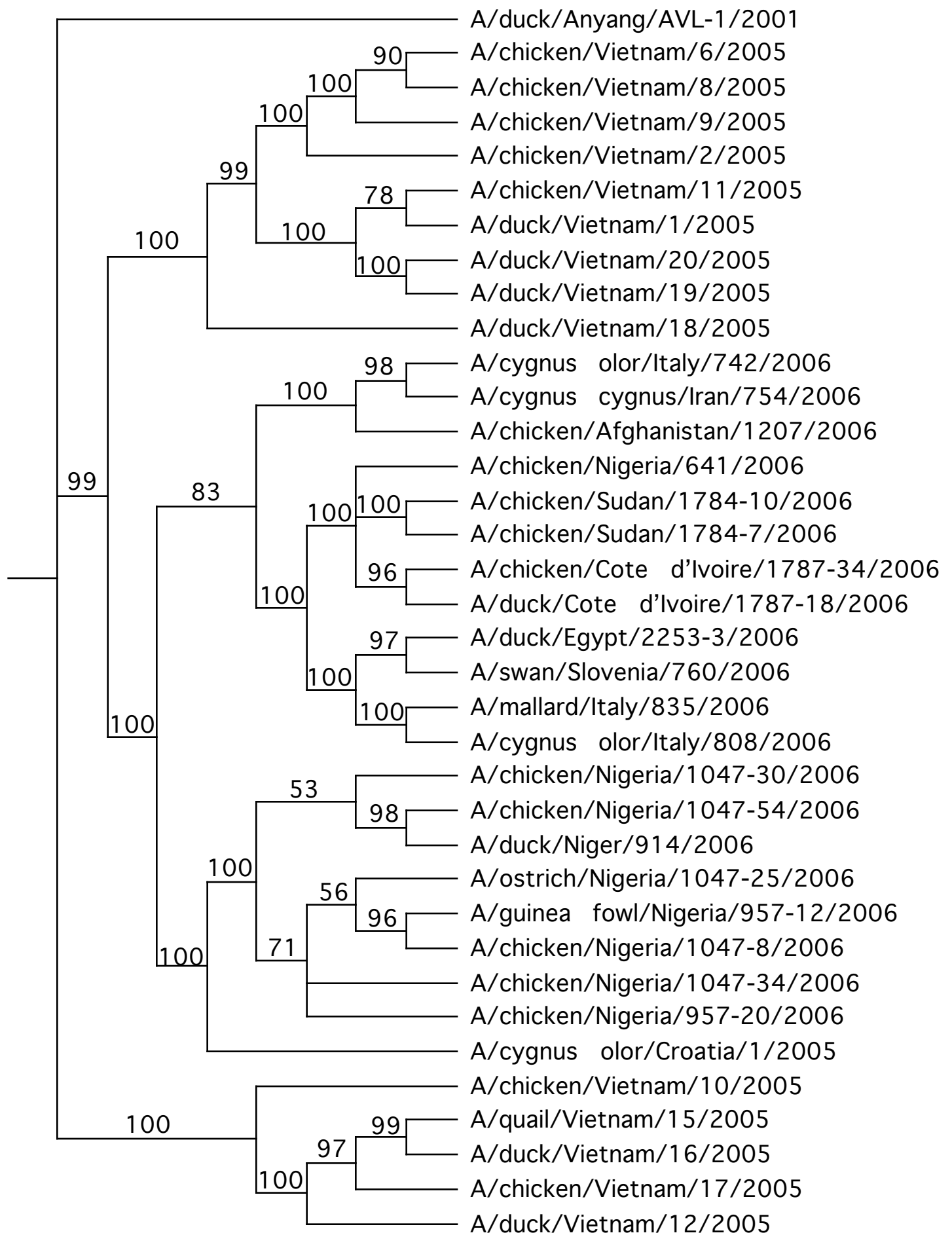

Supplement: Appendix Figure 2 — Consensus of 1,000 neighbor-joining bootstrap replicates as calculated by PAUP 4.0b10 (Swofford DL. PAUP*: Phylogenetic Analysis Using Parsimony [and Other Methods]. Sunderland [MA]: Sinauer Associates; 2002) on 35 of our new 36 isolates, leaving aside the reassortant strain A/Ck/Nigeria/1047 62/2006, which-because of its multilineage ancestry-cannot be placed exclusively in either EMA clade 1 or 2. Each isolate was represented by the concatenation of the alignments of its 8 RNA segments. These bootstrap values are used in the labeling of Figure 1 in the main text. [file 07-0013_appF2-s2.pdf]
