# Supplementary material for: Genome Analysis Linking Recent European and African Influenza (H5N1) Viruses
Source: Emerg Infect Dis. 2007 May;13(5):713–8. doi: 10.3201/eid1305.070013 (PMC2432181; doi:10.3201/eid1305.070013)
Supplement: Appendix Figure 3 — Phylogenetic tree relating hemagglutinin (HA) segments of 589 avian, feline, and human influenza A (H5N1) viruses. This figure is an enlarged view of Figure 2A in the main text. The tree includes all human-derived HA segments deposited in GenBank since the year 2000 that are at least 1000 nt in length (82 sequences) as well as all avian and feline sequences since the year 2000 that are at least 1500 nt in length (4 feline and 503 avian sequences). The sequences were aligned with MUSCLE (Edgar RC. MUSCLE: a multiple sequence alignment method with reduced time and space complexity. BMC Bioinformatics. 2004;5:113), and the tree was created with the neighbor joining algorithm of PAUP* version 4.0b10 (Swofford DL. PAUP*: Phylogenetic Analysis Using Parsimony [and Other Methods]. Sunderland [MA]: Sinauer Associates; 2002) using the F84 distance between the nucleotide sequences and the default parameters. The scale bar in the upper left indicates an F84 distance of 0.01. Human isolates in the figure are red. The V3 clade is pink. The 36 newly sequenced avian isolates are colored according to the region from which they were obtained (blue, Africa; orange, Europe; purple, Middle East; and greenish blue, Vietnam). The isolate A/chicken/Nigeria/1047_62/2006 that has undergone reassortment is marked with an arrow. [file 07-0013_appF3-s3.pdf]

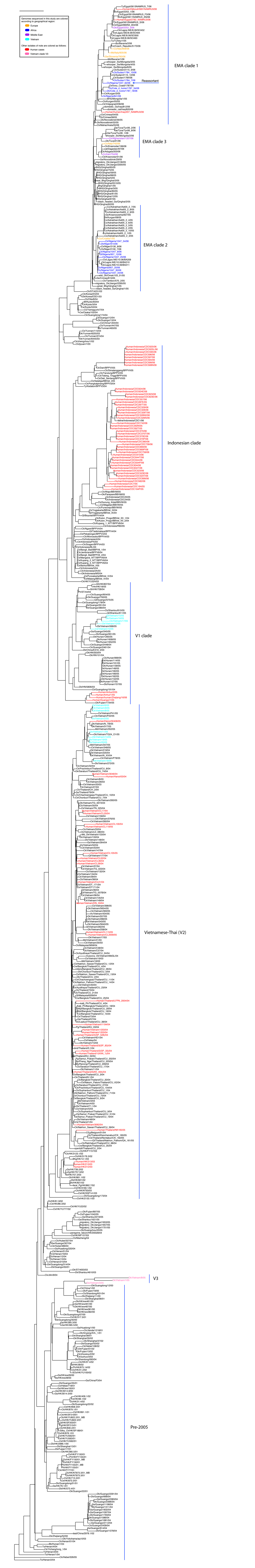

Genomes sequenced in this study are colored according to geographical region:
Europe
Africa
Middle East
Vietnam
Other isolates of note are colored as follows:
Human cases
Vietnam clade V3
EMA clade 1
Reassortant
EMA clade 3
EMA clade 2
Indonesian clade
V1 clade
Vietnamese-Thai (V2)
V3
Pre-2005
