# Supplementary material for: Genome Analysis Linking Recent European and African Influenza (H5N1) Viruses
Source: Emerg Infect Dis. 2007 May;13(5):713–8. doi: 10.3201/eid1305.070013 (PMC2432181; doi:10.3201/eid1305.070013)
Supplement: Appendix Table — Isolate name [file 07-0013_appT-s4.pdf]

Appendix Table.

Isolate name	PB2	PB1	PA	HA	NP	NA	M	NS
A/bar-headed goose/Qinghai/12	DQ095761	DQ095741	DQ095721	DQ095621	DQ095681	DQ095661	DQ095641	DQ095701
A/bar-headed goose/Qinghai/5	DQ095757	DQ095737	DQ095717	DQ095617	DQ095677	DQ095657	DQ095637	DQ095697
A/bar-headed goose/Qinghai/59	DQ095752	DQ095732	DQ095712	DQ095612	DQ095672	DQ095652	DQ095632	DQ095692
A/bar-headed goose/Qinghai/60	DQ095755	DQ095695	DQ095715	DQ095615	DQ095675	DQ095615	DQ095635	DQ095695
A/bar-headed goose/Qinghai/61	DQ095758	DQ095738	DQ095718	DQ095618	DQ095678	DQ095658	DQ095638	DQ095698
A/bar-headed goose/Qinghai/62	DQ095760	DQ095740	DQ095720	DQ095620	DQ095640	DQ095660	DQ095640	DQ095700
A/bar-headed goose/Qinghai/65	DQ095762	DQ095742	DQ095722	DQ095622	DQ095682	DQ095662	DQ095642	DQ095702
A/bar-headed goose/Qinghai/67	DQ095763	DQ095743	DQ095723	DQ095623	DQ095683	DQ095663	DQ095643	DQ095703
A/bar-headed goose/Qinghai/68	DQ095753	DQ095733	DQ095713	DQ095613	DQ095673	DQ095653	DQ095633	DQ095693
A/bar-headed goose/Qinghai/75	DQ095759	DQ095739	DQ095719	DQ095619	DQ095679	DQ095659	DQ095639	DQ095699
A/black-headed goose/Qinghai/1	DQ100542	DQ100546	DQ100550	DQ100554	DQ100558	DQ100562	DQ100566	DQ100570
A/black-headed goose/Qinghai/2	DQ100543	DQ100547	DQ100551	DQ100555	DQ100559	DQ100563	DQ100567	DQ100571
A/black-headed gull/Qinghai/1	DQ100544	DQ100548	DQ100552	DQ100556	DQ100560	DQ100564	DQ100568	DQ100572
A/brown headed gull/Qinghai/3	DQ095756	DQ095736	DQ095716	DQ095616	DQ095676	DQ095656	DQ095636	DQ095696
A/cat Germany/606/2006	DQ643980	DQ643979	DQ643981	DQ643982	DQ643983	DQ643984	DQ643985	DQ643986
A/chicken/Afghanistan/1207/2006	CY016794	CY016793	CY016792	CY016787	CY016790	CY016789	CY016788	CY016791
A/chicken/Cote d'Ivoire/1787/34	CY016818	CY016817	CY016816	CY016811	CY016814	CY016813	CY016812	CY016815
A/chicken/Crimea/08/2005	DQ650670	DQ650669	DQ650668	DQ650663	DQ650666	DQ650665	DQ650664	DQ650667
A/chicken/Fujian/1042	DQ320810	DQ321271	DQ321205	DQ320876	DQ321073	DQ321008	DQ320942	DQ321139
A/chicken/Kurgan/3	DQ449639	DQ449638	DQ449637	DQ449632	DQ449635	DQ449634	DQ449633	DQ449636
A/chicken/Nigeria/1047/30/2006	CY016946	CY016945	CY016944	CY016939	CY016942	CY016941	CY016940	CY016943
A/chicken/Nigeria/1047/34/2006	CY016954	CY016953	CY016952	CY016947	CY016950	CY016949	CY016948	CY016951
A/chicken/Nigeria/1047/54/2006	CY016930	CY016929	CY016928	CY016923	CY016926	CY016925	CY016924	CY016927
A/chicken/Nigeria/1047/8/2006	CY016914	CY016913	CY016912	CY016907	CY016910	CY016909	CY016908	CY016911
A/chicken/Nigeria/641/2006	CY016283	CY016282	CY016281	CY016276	CY016279	CY016278	CY016277	CY016280

A/chicken/Nigeria/957/20/2006	CY016291	CY016290	CY016289	CY016284	CY016287	CY016286	CY016285	CY016288
A/chicken/Shantou/810	DQ095766	DQ095746	DQ095726	DQ095626	DQ095686	DQ095666	DQ095646	DQ095706
A/chicken/Sudan/1784/10/2006	CY016307	CY016306	CY016305	CY016300	CY016303	CY016302	CY016301	CY016304
A/chicken/Sudan/1784/7/2006	CY016299	CY016298	CY016297	CY016292	CY016295	CY016294	CY016293	CY016296
A/Cygnus cygnus/Iran/754/2006	CY016786	CY016785	CY016784	CY016779	CY016782	CY016781	CY016780	CY016783
A/Cygnus olor/Astrakhan/05201	DQ389161	DQ394578	DQ394579	DQ389158	DQ394577	DQ389159	DQ394576	DQ389160
A/Cygnus olor/Astrakhan/05202	DQ343506	DQ343505	DQ343504	DQ343502	DQ359694	DQ343503	DQ359692	DQ359693
A/Cygnus olor/Astrakhan/05203	DQ358750	DQ358749	DQ358748	DQ358746	DQ358751	DQ358747	DQ358739	DQ358752
A/Cygnus olor/Astrakhan/05204	DQ363916	DQ363915	DQ363917	DQ363918	DQ363929	DQ363919	DQ363925	DQ363926
A/Cygnus olor/Astrakhan/05205	DQ365011	DQ365008	DQ365007	DQ365004	DQ365006	DQ365005	DQ365009	DQ365010
A/Cygnus olor/Astrakhan/05206	DQ365001	DQ365000	DQ364999	DQ364996	DQ364998	DQ364997	DQ365002	DQ365003
A/Cygnus olor/Astrakhan/05207	DQ363921	DQ363920	DQ363922	DQ363923	DQ363930	DQ363924	DQ363928	DQ363927
A/Cygnus olor/Astrakhan/05208	DQ386305	DQ386304	DQ399537	DQ399540	DQ399539	DQ399541	DQ399542	DQ399538
A/Cygnus olor/Astrakhan/05209	DQ399543	DQ406738	DQ406737	DQ399547	DQ399545	DQ399546	DQ400912	DQ399544
A/Cygnus olor/Astrakhan/05210	DQ434890	DQ423612	DQ434891	DQ434889	DQ440579	DQ440579	DQ434888	DQ434887
A/Cygnus olor/Croatia/1/2005	CY016826	CY016825	CY016824	CY016819	CY016822	CY016821	CY016820	CY016823
A/Cygnus olor/Italy/742/2006	DQ533586	DQ533585	DQ533584	DQ412997	DQ533582	DQ533581	DQ533580	DQ533583
A/Cygnus olor/Italy/808/2006	tigr/ 1101672593770	tigr/ 1101672593770	tigr/ 1101672593770	tigr/ 1101672593770	tigr/ 1101672593770	tigr/ 1101672593770	tigr/ 1101672593770	tigr/ 1101672593770
A/Djibouti/5691NAMRU3/2006				DQ666146				
A/duck/Cote d'Ivoire/1787/18/2006	CY016810	CY016809	CY016808	CY016803	CY016806	CY016805	CY016804	CY016807
A/duck/Egypt/2253/3/2006	CY016906	CY016905	CY016904	DQ862002	CY016902	CY016901	CY016900	CY016903
A/duck/Fujian/897	DQ320809	DQ321270	DQ321204	DQ320875	DQ321072	DQ321007	DQ320941	DQ321138
A/duck/Hunan/160	DQ320841	DQ321302	DQ321236	DQ320907	DQ321104	DQ321039	DQ320973	DQ321170
A/duck/Kurgan/08/2005	DQ449647	DQ449646	DQ449645	DQ449640	DQ449643	DQ449642	DQ449641	DQ449644
A/duck/Niger/914/2006	CY017034	CY017033	CY017032	DQ659113	CY017030	CY017029	CY017028	CY017031
A/duck/Novosibirsk/56	DQ232608	DQ232606	DQ234076	DQ230522	DQ232610	DQ230524	DQ234078	DQ234074
A/Egypt/2782/NAMRU3/2006				DQ464377				

A/Environment/Qinghai/31/2005	DQ320856	DQ321317	DQ321251	DQ320922	DQ321119	DQ321054	DQ320988	DQ321185
A/goose/Shantou/1621	DQ095768	DQ095748	DQ095728	DQ095628	DQ095688	DQ095668	DQ095648	DQ095708
A/goose/Shantou/2216	DQ320849	DQ321310	DQ321244	DQ320915	DQ321112	DQ321047	DQ320981	DQ321178
A/great black-headed gull/Qinghai	DQ095754	DQ095734	DQ095714	DQ095614	DQ095674	DQ095654	DQ095634	DQ095694
A/grebe/Novosibirsk/29	DQ232607	DQ232605	DQ234075	DQ230521	DQ232609	DQ230523	DQ234077	DQ234073
A/grebe/Tyva/Tyv06/1/2006	DQ914807	DQ914810	DQ978999	DQ914808	DQ916293	DQ914809	DQ914805	DQ914806
A/grebe/Tyva/Tyv06/2/06	DQ852607	DQ852606	DQ852603	DQ852600	DQ852602	DQ852601	DQ852604	DQ852605
A/Grebe/Tyva/Tyv06/8/2006	DQ863510	DQ863509	DQ863508	DQ863503	DQ863506	DQ863507	DQ863504	DQ863505
A/guinea fowl/Nigeria/957/12	CY017186	CY017185	CY017184	CY017179	CY017182	CY017181	CY017180	CY017183
A/human/Iraq/207/NAMRU3/2006				DQ435202				
A/mallard/Italy/835/2006	CY016802	CY016801	CY016800	DQ449031	CY016798	CY016797	CY016796	CY016799
A/migratory duck/Jiangxi/1653	DQ320850	DQ321311	DQ321245	DQ320916	DQ321113	DQ321048	DQ320982	DQ321179
A/migratory duck/Jiangxi/1657	DQ320851	DQ321312	DQ321246	DQ320917	DQ321114	DQ321049	DQ320983	DQ321180
A/migratory duck/Jiangxi/1701	DQ320852	DQ321313	DQ321247	DQ320918	DQ321115	DQ321050	DQ320984	DQ321181
A/ostrich/Nigeria/1047/25/2006	CY016922	CY016921	CY016920	CY016915	CY016918	CY016917	CY016916	CY016919
A/quail/Shantou/911	DQ095767	DQ095747	DQ095727	DQ095627	DQ095687	DQ095667	DQ095647	DQ095707
A/swan/Germany/R65/2006	DQ464357	DQ464361	DQ464360	DQ464354	DQ464359	DQ464355	DQ464356	DQ464358
A/swan/Slovenia/760/2006	CY017050	CY017049	CY017048	CY017043	CY017046	CY017045	CY017044	CY017047
A/whooper swan/Mongolia/2/06	AB264769	AB264770	AB263751	AB263752	AB263753	AB265202	AB265203	AB265204
A/whooper swan/Mongolia/3/05	AB239307	AB239308	AB239309	AB233320	AB239310	AB239311	AB239312	AB239313
A/whooper swan/Mongolia/4/05	AB239314	AB239315	AB239316	AB233321	AB239317	AB239318	AB239319	AB239320
A/whooper swan/Mongolia/6/05	AB239321	AB239322	AB239323	AB233322	AB239324	AB239325	AB239326	AB239327
