# Supplementary material for: Genome Analysis Linking Recent European and African Influenza (H5N1) Viruses
Source: Emerg Infect Dis. 2007 May;13(5):713–8. doi: 10.3201/eid1305.070013 (PMC2432181; doi:10.3201/eid1305.070013)
Supplement: Technical Appendix 1 — POSITION 627 IN PB2 PROTEIN FOR ALL 420 SEQUENCES AVAILABLE FROM 2001-2006 AS OF November 2006 [file 07-0013_Techapp1-s5.pdf]

POSITION 627 IN PB2 PROTEIN FOR ALL 420 SEQUENCES AVAILABLE FROM 2001-2006 AS OF November 2006
Column 1: amino acid, either E (glutamic acid) or K (lysine). All 64 lysines appear at the bottom of the file. Column 2 is the isolate identifier, and the 36 new sequences added by this study have a label in column 3.

E A/bird/Thailand/3.1/2004(H5N1)
E A/chicken/Ayutthaya/Thailand/CU-23/04(H5N1)
E A/chicken/Bangli_Bali/BBPV6-1/2004(H5N1)
E A/chicken/Bangli_Bali/BPPV6-2/2004(H5N1)
E A/chicken/Dairi/BPPVI/2005(H5N1)
E A/chicken/Deli_Serdang/BPPVI/2005(H5N1)
E A/chicken/Fujian/10039/2005(H5N1)
E A/chicken/Fujian/1042/2005(H5N1)
E A/chicken/Fujian/10567/2005(H5N1)
E A/chicken/Fujian/11933/2005(H5N1)
E A/chicken/Fujian/12239/2005(H5N1)
E A/chicken/Fujian/584/2006(H5N1)
E A/chicken/Fujian/9821/2005(H5N1)
E A/chicken/Guangdong/174/04(H5N1)
E A/chicken/Guangdong/178/04(H5N1)
E A/chicken/Guangdong/191/04(H5N1)
E A/chicken/Guangxi/1212/2006(H5N1)
E A/chicken/Guangxi/12/2004(H5N1)
E A/chicken/Guangxi/1951/2006(H5N1)
E A/chicken/Guangxi/3154/2005(H5N1)
E A/chicken/Guangxi/3791/2005(H5N1)
E A/chicken/Guangxi/463/2006(H5N1)
E A/chicken/Guangxi/4989/2005(H5N1)
E A/chicken/Guangxi/604/2005(H5N1)
E A/chicken/Guangxi/683/2006(H5N1)
E A/chicken/Guiyang/1655/2006(H5N1)
E A/chicken/Guiyang/2147/2005(H5N1)
E A/chicken/Guiyang/2173/2005(H5N1)
E A/chicken/Guiyang/237/2006(H5N1)
E A/chicken/Guiyang/29/2006(H5N1)
E A/chicken/Guiyang/3055/2005(H5N1)
E A/chicken/Guiyang/3570/2005(H5N1)
E A/chicken/Guiyang/3721/2005(H5N1)
E A/chicken/Guiyang/3923/2005(H5N1)
E A/chicken/Guiyang/4059/2005(H5N1)
E A/chicken/Guiyang/441/2006(H5N1)
E A/chicken/Gunung_Kidal/BBVW/2005(H5N1)
E A/chicken/Hebei/108/02(H5N1)
E A/chicken/Hebei/326/2005(H5N1)
E A/chicken/Hebei/718/2001(H5N1)
E A/chicken/Henan/1/2004(H5N1)
E A/chicken/Henan/12/2004(H5N1)
E A/chicken/Henan/13/2004(H5N1)
E A/chicken/Henan/16/2004(H5N1)
E A/chicken/Henan/wu/2004(H5N1)
E A/chicken/Hong_Kong/282/2006(H5N1)
E A/chicken/Hong_Kong/947/2006(H5N1)
E A/Chicken/HongKong/FY150/01(H5N1)
E A/Chicken/HongKong/FY150/01-MB(H5N1)
E A/Chicken/HongKong/NT873.3/01(H5N1)
E A/Chicken/HongKong/NT873.3/01-MB(H5N1)
E A/Chicken/HongKong/YU562/01(H5N1)
E A/Chicken/HongKong/YU822.2/01(H5N1)
E A/Chicken/HongKong/YU822.2/01-MB(H5N1)
E A/chicken/Hubei/327/2004(H5N1)
E A/chicken/Hubei/489/2004(H5N1)
E A/chicken/Hubei/wf/2002(H5N1)
E A/chicken/Hubei/wn/2003(H5N1)
E A/chicken/Hubei/wo/2003(H5N1)
E A/chicken/Hunan/999/2005(H5N1)
E A/chicken/Indonesia/CDC24/2005(H5N1)
E A/chicken/Indonesia/CDC25/2005(H5N1)
E A/chicken/Jiangsu/cz1/2002(H5N1)
E A/chicken/Jilin/9/2004(H5N1)
E A/chicken/Jilin/ha/2003(H5N1)
E A/chicken/Jilin/hd/2002(H5N1)
E A/chicken/Jilin/he/2002(H5N1)
E A/chicken/Jilin/hf/2002(H5N1)
E A/chicken/Jilin/hg/2002(H5N1)
E A/chicken/Jilin/hj/2003(H5N1)
E A/chicken/Jilin/hk/2004(H5N1)
E A/chicken/Jilin/hl/2004(H5N1)
E A/chicken/Jilin/hm/2003(H5N1)
E A/chicken/Jilin/xw/2003(H5N1)
E A/chicken/Korea/ES/03(H5N1)
E A/chicken/Kulon_Progo/BBVet-XII-1/2004(H5N1)

E A/chicken/Kulon_Progo/BBVet-XII-2/2004(H5N1)
E A/chicken/Kulon_Progo/BBVW/2005(H5N1)
E A/chicken/Kupang-2-NTT/BPPV6/2004(H5N1)
E A/chicken/Kupang-3-NTT/BPPV6/2004(H5N1)
E A/chicken/Kyoto/3/2004(H5N1)
E A/chicken/Magetan/BBVW/2005(H5N1)
E A/chicken/Malang/BBVet-IV/2004(H5N1)
E A/chicken/Mangarai-NTT/BPPV6/2004(H5N1)
E A/chicken/Nakorn-Patom/Thailand/CU-K2/2004(H5N1)
E A/chicken/Oita/8/2004(H5N1)
E A/chicken/Purwakarta/BBVet-IV/2004(H5N1)
E A/chicken/Purworejo/BBVW/2005(H5N1)
E A/chicken/Salatiga/BBVet-I/2005(H5N1)
E A/chicken/Shantou/1233/2006(H5N1)
E A/chicken/Shantou/3840/2006(H5N1)
E A/chicken/Shantou/3923/2006(H5N1)
E A/chicken/Shanxi/2/2006(H5N1)
E A/chicken/Simalanggang/BPPVI/2005(H5N1)
E A/chicken/Tarutung/BPPVI/2005(H5N1)
E A/chicken/Tebing_Tinggi/BPPVI/2005(H5N1)
E A/chicken/Thailand/Kanchanaburi/CK-160/2005(H5N1)
E A/chicken/Thailand/Nontaburi/CK-162/2005(H5N1)
E A/chicken/Thailand/NP-172/2006(H5N1)
E A/chicken/Thailand/PC-168/2006(H5N1)
E A/chicken/Thailand/PC-170/2006(H5N1)
E A/chicken/Tula/Russia/Oct-5/2005(H5N1)
E A/chicken/Viet_Nam/10/2005(H5N1)
E A/chicken/Viet_Nam/11/2005(H5N1)
E A/chicken/Viet_Nam/17/2005(H5N1)
E A/chicken/Viet_Nam/2/2005(H5N1)
E A/chicken/Viet_Nam/6/2005(H5N1)
E A/chicken/Viet_Nam/8/2005(H5N1)
E A/chicken/Viet_Nam/9/2005(H5N1)
E A/chicken/Viet_Nam/AG-010/2004(H5N1)
E A/chicken/Vietnam/C58/04(H5N1)
E A/chicken/Viet_Nam/CT-018/2004(H5N1)
E A/chicken/Viet_Nam/DN-045/2004(H5N1)
E A/chicken/Viet_Nam/DT-015/2004(H5N1)
E A/chicken/Viet_Nam/DT-171/2004(H5N1)
E A/chicken/Viet_Nam/HCM-022/2004(H5N1)
E A/chicken/Viet_Nam/LA-024/2004(H5N1)
E A/chicken/Viet_Nam/LD-080/2004(H5N1)
E A/chicken/Viet_Nam/TG-023/2004(H5N1)
E A/chicken/Viet_Nam/TN-025/2004(H5N1)
E A/chicken/Viet_Nam/VL-008/2004(H5N1)
E A/chicken/Wajo/BBVM/2005(H5N1)
E A/chicken/Yamaguchi/7/2004(H5N1)
E A/chicken/Yogjakarta/BBVet-IX/2004(H5N1)
E A/Ck/Indonesia/BL/2003(H5N1)
E A/Ck/Indonesia/PA/2003(H5N1)
E A/Ck/Thailand/9.1/2004(H5N1)
E A/Ck/Viet_Nam/33/2004(H5N1)
E A/Ck/Viet_Nam/35/2004(H5N1)
E A/Ck/Viet_Nam/36/2004(H5N1)
E A/Ck/Viet_Nam/37/2004(H5N1)
E A/Ck/Viet_Nam/38/2004(H5N1)
E A/Ck/Viet_Nam/39/2004(H5N1)
E A/Ck/Viet_Nam/C57/2004(H5N1)
E A/common_magpie/Hong_Kong/2125/2006(H5N1)
E A/common_magpie/Hong_Kong/2256/2006(H5N1)
E A/common_magpie/Hong_Kong/3033/2006(H5N1)
E A/common_magpie/Hong_Kong/645/2006(H5N1)
E A/crested_eagle/Belgium/01/2004(H5N1)
E A/crested_myna/Hong_Kong/540/2006(H5N1)
E A/crow/Kyoto/53/2004(H5N1)
E A/crow/Osaka/102/2004(H5N1)
E A/Cygnus_olor/Astrakhan/Ast05-2-4/2005(H5N1)
E A/Dk/Indonesia/MS/2004(H5N1)
E A/Dk/Thailand/71.1/2004(H5N1)
E A/Dk/Viet_Nam/11/2004(H5N1)
E A/Duck/Anyang/AVL-1/2001(H5N1)
E A/duck/China/E319-2/03(H5N1)
E A/duck/Fujian/01/2002(H5N1)
E A/duck/Fujian/10160/2005(H5N1)
E A/duck/Fujian/10389/2005(H5N1)
E A/duck/Fujian/10934/2005(H5N1)
E A/duck/Fujian/11094/2005(H5N1)
E A/duck/Fujian/11311/2005(H5N1)
E A/duck/Fujian/12032/2005(H5N1)
E A/duck/Fujian/13/2002(H5N1)
E A/duck/Fujian/17/2001(H5N1)

E A/duck/Fujian/668/2006(H5N1)
E A/duck/Fujian/671/2006(H5N1)
E A/duck/Fujian/720/2006(H5N1)
E A/duck/Fujian/897/2005(H5N1)
E A/duck/Fujian/9651/2005(H5N1)
E A/duck/Fujian/9713/2005(H5N1)
E A/duck/Guangdong/01/2001(H5N1)
E A/duck/Guangdong/173/04(H5N1)
E A/duck/Guangdong/22/2002(H5N1)
E A/duck/Guangxi/1258/2006(H5N1)
E A/duck/Guangxi/1311/2004(H5N1)
E A/duck/Guangxi/13/2004(H5N1)
E A/duck/Guangxi/1378/2004(H5N1)
E A/duck/Guangxi/1436/2006(H5N1)
E A/duck/Guangxi/150/2006(H5N1)
E A/duck/Guangxi/1550/2006(H5N1)
E A/duck/Guangxi/1586/2004(H5N1)
E A/duck/Guangxi/1681/2004(H5N1)
E A/duck/Guangxi/1793/2004(H5N1)
E A/duck/Guangxi/1830/2006(H5N1)
E A/duck/Guangxi/2143/2006(H5N1)
E A/duck/Guangxi/22/2001(H5N1)
E A/duck/Guangxi/2291/2004(H5N1)
E A/duck/Guangxi/2396/2004(H5N1)
E A/duck/Guangxi/2775/2005(H5N1)
E A/duck/Guangxi/288/2006(H5N1)
E A/duck/Guangxi/2926/2005(H5N1)
E A/duck/Guangxi/3085/2005(H5N1)
E A/duck/Guangxi/3364/2005(H5N1)
E A/duck/Guangxi/351/2004(H5N1)
E A/duck/Guangxi/35/2001(H5N1)
E A/duck/Guangxi/3548/2005(H5N1)
E A/duck/Guangxi/3714/2005(H5N1)
E A/duck/Guangxi/3741/2005(H5N1)
E A/duck/Guangxi/3819/2005(H5N1)
E A/duck/Guangxi/392/2006(H5N1)
E A/duck/Guangxi/4016/2005(H5N1)
E A/duck/Guangxi/4184/2005(H5N1)
E A/duck/Guangxi/4196/2005(H5N1)
E A/duck/Guangxi/4428/2005(H5N1)
E A/duck/Guangxi/4665/2005(H5N1)
E A/duck/Guangxi/4830/2005(H5N1)
E A/duck/Guangxi/50/2001(H5N1)
E A/duck/Guangxi/5075/2005(H5N1)
E A/duck/Guangxi/5165/2005(H5N1)
E A/duck/Guangxi/5270/2005(H5N1)
E A/duck/Guangxi/53/2002(H5N1)
E A/duck/Guangxi/5457/2005(H5N1)
E A/duck/Guangxi/619/2006(H5N1)
E A/duck/Guangxi/668/2004(H5N1)
E A/duck/Guangxi/744/2006(H5N1)
E A/duck/Guangxi/793/2005(H5N1)
E A/duck/Guangxi/804/2006(H5N1)
E A/duck/Guangxi/89/2006(H5N1)
E A/duck/Guangxi/951/2005(H5N1)
E A/duck/Guangxi/xa/2001(H5N1)
E A/duck/Guangzhou/20/2005(H5N1)
E A/duck/Guiyang/1081/2006(H5N1)
E A/duck/Guiyang/1588/2006(H5N1)
E A/duck/Guiyang/1722/2006(H5N1)
E A/duck/Guiyang/2231/2005(H5N1)
E A/duck/Guiyang/293/2006(H5N1)
E A/duck/Guiyang/3009/2005(H5N1)
E A/duck/Guiyang/3242/2005(H5N1)
E A/duck/Guiyang/3834/2005(H5N1)
E A/duck/Guiyang/3996/2005(H5N1)
E A/duck/Guiyang/497/2006(H5N1)
E A/duck/Hokkaido/Vac-1/04(H5N1)
E A/duck/Hong_Kong/821/02(H5N1)
E A/duck/Hubei/wg/2002(H5N1)
E A/duck/Hubei/wp/2003(H5N1)
E A/duck/Hubei/wq/2003(H5N1)
E A/duck/Hunan/1204/2006(H5N1)
E A/duck/Hunan/1265/2005(H5N1)
E A/duck/Hunan/1608/2005(H5N1)
E A/duck/Hunan/1652/2005(H5N1)
E A/duck/Hunan/324/2006(H5N1)
E A/duck/Hunan/344/2006(H5N1)
E A/duck/Hunan/5106/2005(H5N1)
E A/duck/Hunan/5152/2005(H5N1)
E A/duck/Hunan/5472/2005(H5N1)

E A/duck/Hunan/856/2006(H5N1)
E A/duck/Hunan/988/2006(H5N1)
E A/duck/Korea/ESD1/03(H5N1)
E A/duck/Kurgan/08/2005(H5N1)
E A/duck/Novosibirsk/02/05(H5N1)
E A/duck/Parepare/BBVM/2005(H5N1)
E A/duck/Shandong/093/2004(H5N1)
E A/duck/Shanghai/08/2001(H5N1)
E A/duck/Shanghai/13/2001(H5N1)
E A/duck/Shanghai/35/2002(H5N1)
E A/duck/Shanghai/37/2002(H5N1)
E A/duck/Shanghai/38/2001(H5N1)
E A/duck/Shanghai/xj/2002(H5N1)
E A/duck/Shantou/13323/2005(H5N1)
E A/duck/Shantou/4610/2003(H5N1)
E A/duck/Vietnam/1/2005(H5N1)
E A/duck/Viet_Nam/1/2005(H5N1)
E A/duck/Viet_Nam/12/2005(H5N1)
E A/duck/Viet_Nam/18/2005(H5N1)
E A/duck/Viet_Nam/19/2005(H5N1)
E A/duck/Viet_Nam/20/2005(H5N1)
E A/duck/Vietnam/8/05(H5N1)
E A/duck/Viet_Nam/CM-V7/2004(H5N1)
E A/duck/Viet_Nam/TG-007A/2004(H5N1)
E A/duck/Viet_Nam/TV-V2/2004(H5N1)
E A/duck/Yokohama/aq10/2003(H5N1)
E A/duck/Yunnan/4400/2005(H5N1)
E A/duck/Yunnan/4589/2005(H5N1)
E A/duck/Yunnan/5133/2005(H5N1)
E A/duck/Yunnan/5236/2005(H5N1)
E A/duck/Yunnan/5251/2005(H5N1)
E A/duck/Yunnan/5820/2005(H5N1)
E A/duck/Yunnan/5877/2005(H5N1)
E A/duck/Yunnan/6332/2005(H5N1)
E A/duck/Yunnan/6607/2005(H5N1)
E A/duck/Zhejiang/bj/2002(H5N1)
E A/egret/Hong_Kong/757.2/03(H5N1)
E A/Gf/HK/38/2002(H5N1)
E A/goose/Fujian/bb/2003(H5N1)
E A/goose/Guangdong/xb/2001(H5N1)
E A/goose/Guangxi/1097/2004(H5N1)
E A/goose/Guangxi/1198/2004(H5N1)
E A/goose/Guangxi/1458/2006(H5N1)
E A/goose/Guangxi/1633/2006(H5N1)
E A/goose/Guangxi/1832/2004(H5N1)
E A/goose/Guangxi/1898/2006(H5N1)
E A/goose/Guangxi/2112/2004(H5N1)
E A/goose/Guangxi/224/2006(H5N1)
E A/goose/Guangxi/2383/2004(H5N1)
E A/goose/Guangxi/3017/2005(H5N1)
E A/goose/Guangxi/3316/2005(H5N1)
E A/goose/Guangxi/345/2005(H5N1)
E A/goose/Guangxi/4289/2005(H5N1)
E A/goose/Guangxi/4513/2005(H5N1)
E A/goose/Guangxi/532/2006(H5N1)
E A/goose/Guangxi/5414/2005(H5N1)
E A/goose/Guangxi/582/2006(H5N1)
E A/goose/Guangxi/914/2004(H5N1)
E A/goose/Guiyang/1461/2006(H5N1)
E A/goose/Guiyang/1636/2006(H5N1)
E A/goose/Guiyang/1794/2006(H5N1)
E A/goose/Guiyang/337/2006(H5N1)
E A/goose/Guiyang/3422/2005(H5N1)
E A/goose/Guiyang/4030/2005(H5N1)
E A/goose/Guiyang/4180/2005(H5N1)
E A/goose/Guiyang/538/2006(H5N1)
E A/goose/Guiyang/765/2006(H5N1)
E A/goose/Jilin/hb/2003(H5N1)
E A/goose/Shantou/18442/2005(H5N1)
E A/goose/Shantou/2086/2006(H5N1)
E A/goose/Shantou/2216/2005(H5N1)
E A/goose/Shantou/239/2006(H5N1)
E A/goose/Shantou/3265/2006(H5N1)
E A/goose/Shantou/3295/2006(H5N1)
E A/goose/Shantou/3624/2006(H5N1)
E A/goose/Vietnam/3/05(H5N1)
E A/goose/Yunnan/3315/2005(H5N1)
E A/goose/Yunnan/3644/2005(H5N1)
E A/goose/Yunnan/3720/2005(H5N1)
E A/goose/Yunnan/4129/2005(H5N1)
E A/goose/Yunnan/4494/2005(H5N1)

E A/goose/Yunnan/4804/2005(H5N1)
E A/goose/Yunnan/5299/2005(H5N1)
E A/goose/Yunnan/5539/2005(H5N1)
E A/goose/Yunnan/6027/2005(H5N1)
E A/goose/Yunnan/6368/2005(H5N1)
E A/house_crow/Hong_Kong/2648/2006(H5N1)
E A/Japanese_white-eye/Hong_Kong/1038/2006(H5N1)
E A/large-billed_crow/Hong_Kong/2512/2006(H5N1)
E A/little_egret/Hong_Kong/718/2006(H5N1)
E A/mallard/Guangxi/wt/2004(H5N1)
E A/mallard/Italy/835/2006(H5N1)
E A/migratory_duck/Jiangxi/2300/2005(H5N1)
E A/munia/Hong_Kong/2454/2006(H5N1)
E A/peregrine_falcon/HK/D0028/2004(H5N1)
E A/Pheasant/HongKong/FY155/01(H5N1)
E A/pheasant/Shantou/2239/2006(H5N1)
E A/pigeon/Thailand/KU-03/04(H5N1)
E A/Qa/Thailand/57/2004(H5N1)
E A/quail/Guangxi/575/2005(H5N1)
E A/quail/Thailand/Nakhon_Pathom/QA-161/2005(H5N1)
E A/quail/Viet_Nam/15/2005(H5N1)
E A/quail/Vietnam/36/04(H5N1)
E A/quail/Viet_Nam/TG-007B/2004(H5N1)
E A/quail/Yogjakarta/BBVet-IX/2004(H5N1)
E A/R(duck/Mongolia/54/01-duck/Mongolia/47/01)(H5N1)
E A/robin/Hong_Kong/366/2006(H5N1)
E A/robin/Hong_Kong/75/2006(H5N1)
E A/SCk/HK/YU100/2002(H5N1)
E A/swan/Guangxi/307/2004(H5N1)
E A/Tree_sparrow/Henan/1/2004(H5N1)
E A/Tree_sparrow/Henan/2/2004(H5N1)
E A/Tree_sparrow/Henan/3/2004(H5N1)
E A/Tree_sparrow/Henan/4/2004(H5N1)
E A/white-backed_munia/Hong_Kong/2469/2006(H5N1)
E A/wild_duck/Guangdong/314/2004(H5N1)
E A/wild_duck/Shantou/21498/2005(H5N1)
(H5N1) E A/Ck/HK/31.4/02
(H5N1) E A/Ck/HK/YU777/02
K A/bar-headed_goose/Mongolia/1/05(H5N1)
K A/bar-headed_goose/Qinghai/0510/05(H5N1)
K A/Bar-headed_Goose/Qinghai/12/05(H5N1)
K A/Bar-headed_Goose/Qinghai/5/05(H5N1)
K A/Bar-headed_Goose/Qinghai/59/05(H5N1)
K A/Bar-headed_Goose/Qinghai/60/05(H5N1)
K A/Bar-headed_Goose/Qinghai/61/05(H5N1)
K A/Bar-headed_Goose/Qinghai/62/05(H5N1)
K A/Bar-headed_Goose/Qinghai/65/05(H5N1)
K A/Bar-headed_Goose/Qinghai/67/05(H5N1)
K A/Bar-headed_Goose/Qinghai/68/05(H5N1)
K A/Bar-headed_Goose/Qinghai/75/05(H5N1)
K A/black-headed_goose/Qinghai/1/2005(H5N1)
K A/black-headed_goose/Qinghai/2/2005(H5N1)
K A/black-headed_gull/Qinghai/1/2005(H5N1)
K A/Brown-headed_Gull/Qinghai/3/05(H5N1)
K A/chicken/Afghanistan/1207/2006(H5N1)
K A/chicken/Cote_d'Ivoire/1787-34/2006(H5N1)
K A/chicken/Crimea/08/2005(H5N1)
K A/chicken/Kurgan/05/2005(H5N1)
K A/chicken/Kurgan/3/2005(H5N1)
K A/chicken/Nigeria/1047-30/2006(H5N1)
K A/chicken/Nigeria/1047-34/2006(H5N1)
K A/chicken/Nigeria/1047-54/2006(H5N1)
K A/chicken/Nigeria/1047-62/2006(H5N1)
K A/chicken/Nigeria/1047-8/2006(H5N1)
K A/chicken/Nigeria/641/2006(H5N1)
K A/chicken/Nigeria/641/2006(H5N1)
K A/chicken/Nigeria/957-20/2006(H5N1)
K A/chicken/Sudan/1784-10/2006(H5N1)
K A/chicken/Sudan/1784-7/2006(H5N1)
K A/cygnus_cygnus/Iran/754/2006(H5N1)
K A/Cygnus_olor/Astrakhan/Ast05-2-10/2005(H5N1)
K A/Cygnus_olor/Astrakhan/Ast05-2-1/2005(H5N1)
K A/Cygnus_olor/Astrakhan/Ast05-2-2/2005(H5N1)
K A/Cygnus_olor/Astrakhan/Ast05-2-3/2005(H5N1)
K A/Cygnus_olor/Astrakhan/Ast05-2-5/2005(H5N1)
K A/Cygnus_olor/Astrakhan/Ast05-2-6/2005(H5N1)
K A/Cygnus_olor/Astrakhan/Ast05-2-7/2005(H5N1)
K A/Cygnus_olor/Astrakhan/Ast05-2-8/2005(H5N1)
K A/Cygnus_olor/Astrakhan/Ast05-2-9/2005(H5N1)
K A/cygnus_olor/Croatia/1/2005(H5N1)
K A/cygnus olor/Italy/742/2006(H5N1)

K A/Cygnus_olor/Italy/742/2006(H5N1)
K A/duck/Cote_d'Ivoire/1787-18/2006(H5N1)
K A/duck/Egypt/2253-3/2006(H5N1)
K A/duck/Niger/914/2006(H5N1)
K A/duck/Novosibirsk/56/05(H5N1)
K A/great_black-headed_gull/Qinghai/1/2005(H5N1)
K A/Great_Black-headed_Gull/Qinghai/2/05(H5N1)
K A/grebe/Novosibirsk/29/05(H5N1)
K A/grebe/Tyva/Tyv06-1/06(H5N1)
K A/grebe/Tyva/Tyv06-2/06(H5N1)
K A/Grebe/Tyva/Tyv06-8/2006(H5N1)
K A/guinea_fowl/Nigeria/957-12/2006(H5N1)
K A/Guinea_fowl/Shantou/1341/2006(H5N1)
K A/ostrich/Nigeria/1047-25/2006(H5N1)
K A/Pheasant/HongKong/FY155/01-MB(H5N1)
K A/swan/Astrakhan/Russia/Nov-2/2005(H5N1)
K A/swan/Germany/R65/2006(H5N1)
K A/swan/Slovenia/760/2006(H5N1)
K A/whooper_swan/Mongolia/2/06(H5N1)
K A/whooper_swan/Mongolia/3/05(H5N1)
