# Supplementary material for: Genome Analysis Linking Recent European and African Influenza (H5N1) Viruses
Source: Emerg Infect Dis. 2007 May;13(5):713–8. doi: 10.3201/eid1305.070013 (PMC2432181; doi:10.3201/eid1305.070013)
Supplement: Technical Appendix 2 — 1: CY017194 Influenza A virus (A/duck/Viet Nam/19/2005(H5N1) [file 07-0013_Techapp2-s6.pdf]

1:  CY017194
Influenza A virus (A/duck/Viet Nam/19/2005(H5N1)) segment 1, complete sequence
gi|117571368|gb|CY017194.1|[117571368]

2:  CY017193
Influenza A virus (A/duck/Viet Nam/19/2005(H5N1)) segment 2, complete sequence
gi|117571365|gb|CY017193.1|[117571365]

3:  CY017192
Influenza A virus (A/duck/Viet Nam/19/2005(H5N1)) segment 3, complete sequence
gi|117571363|gb|CY017192.1|[117571363]

4:  CY017191
Influenza A virus (A/duck/Viet Nam/19/2005(H5N1)) segment 8, complete sequence
gi|117571359|gb|CY017191.1|[117571359]

5:  CY017190
Influenza A virus (A/duck/Viet Nam/19/2005(H5N1)) segment 5, complete sequence
gi|117571357|gb|CY017190.1|[117571357]

6:  CY017189
Influenza A virus (A/duck/Viet Nam/19/2005(H5N1)) segment 6, complete sequence
gi|117571355|gb|CY017189.1|[117571355]

7:  CY017188
Influenza A virus (A/duck/Viet Nam/19/2005(H5N1)) segment 7, complete sequence
gi|117571351|gb|CY017188.1|[117571351]

8:  CY017187
Influenza A virus (A/duck/Viet Nam/19/2005(H5N1)) segment 4, complete sequence
gi|117571349|gb|CY017187.1|[117571349]

9:  CY017186
Influenza A virus (A/guinea fowl/Nigeria/957-12/2006(H5N1)) segment 1, complete sequence
gi|117571327|gb|CY017186.1|[117571327]

10:  CY017185
Influenza A virus (A/guinea fowl/Nigeria/957-12/2006(H5N1)) segment 2, complete sequence
gi|117571323|gb|CY017185.1|[117571323]

11:  CY017184
Influenza A virus (A/guinea fowl/Nigeria/957-12/2006(H5N1)) segment 3, complete sequence
gi|117571321|gb|CY017184.1|[117571321]

12:  CY017183
Influenza A virus (A/guinea fowl/Nigeria/957-12/2006(H5N1)) segment 8, complete sequence
gi|117571318|gb|CY017183.1|[117571318]

13:  CY017182
Influenza A virus (A/guinea fowl/Nigeria/957-12/2006(H5N1)) segment 5, complete sequence
gi|117571315|gb|CY017182.1|[117571315]

14:  CY017181
Influenza A virus (A/guinea fowl/Nigeria/957-12/2006(H5N1)) segment 6, complete sequence
gi|117571313|gb|CY017181.1|[117571313]

15:  CY017180
Influenza A virus (A/guinea fowl/Nigeria/957-12/2006(H5N1)) segment 7, complete sequence
gi|117571309|gb|CY017180.1|[117571309]

16:  CY017179
Influenza A virus (A/guinea fowl/Nigeria/957-12/2006(H5N1)) segment 4, complete sequence
gi|117571307|gb|CY017179.1|[117571307]

17: CY017058
Influenza A virus (A/quail/Viet Nam/15/2005(H5N1)) segment 1, complete sequence
gi|116070400|gb|CY017058.1|[116070400]

18: CY017057
Influenza A virus (A/quail/Viet Nam/15/2005(H5N1)) segment 2, complete sequence
gi|116070397|gb|CY017057.1|[116070397]

19: CY017056
Influenza A virus (A/quail/Viet Nam/15/2005(H5N1)) segment 3, complete sequence
gi|116070395|gb|CY017056.1|[116070395]

20: CY017055
Influenza A virus (A/quail/Viet Nam/15/2005(H5N1)) segment 8, complete sequence
gi|116070392|gb|CY017055.1|[116070392]

21: CY017054
Influenza A virus (A/quail/Viet Nam/15/2005(H5N1)) segment 5, complete sequence
gi|116070390|gb|CY017054.1|[116070390]

22: CY017053
Influenza A virus (A/quail/Viet Nam/15/2005(H5N1)) segment 6, complete sequence
gi|116070388|gb|CY017053.1|[116070388]

23: CY017052
Influenza A virus (A/quail/Viet Nam/15/2005(H5N1)) segment 7, complete sequence
gi|116070385|gb|CY017052.1|[116070385]

24: CY017051
Influenza A virus (A/quail/Viet Nam/15/2005(H5N1)) segment 4, complete sequence
gi|116070383|gb|CY017051.1|[116070383]

25: CY017066
Influenza A virus (A/chicken/Viet Nam/17/2005(H5N1)) segment 1, complete sequence
gi|116070381|gb|CY017066.1|[116070381]

26: CY017065
Influenza A virus (A/chicken/Viet Nam/17/2005(H5N1)) segment 2, complete sequence
gi|116070378|gb|CY017065.1|[116070378]

27: CY017064
Influenza A virus (A/chicken/Viet Nam/17/2005(H5N1)) segment 3, complete sequence
gi|116070376|gb|CY017064.1|[116070376]

28: CY017063
Influenza A virus (A/chicken/Viet Nam/17/2005(H5N1)) segment 8, complete sequence
gi|116070373|gb|CY017063.1|[116070373]

29: CY017062
Influenza A virus (A/chicken/Viet Nam/17/2005(H5N1)) segment 5, complete sequence
gi|116070371|gb|CY017062.1|[116070371]

30: CY017061
Influenza A virus (A/chicken/Viet Nam/17/2005(H5N1)) segment 6, complete sequence
gi|116070369|gb|CY017061.1|[116070369]

31: CY017060
Influenza A virus (A/chicken/Viet Nam/17/2005(H5N1)) segment 7, complete sequence
gi|116070366|gb|CY017060.1|[116070366]

32: CY017059
Influenza A virus (A/chicken/Viet Nam/17/2005(H5N1)) segment 4, complete sequence
gi|116070364|gb|CY017059.1|[116070364]

33: CY017035

Influenza A virus (A/cygnus olor/Italy/742/2006(H5N1)) segment 4, complete sequence
gi|116070055|gb|CY017035.1|[116070055]

34: CY017074
Influenza A virus (A/duck/Viet Nam/18/2005(H5N1)) segment 1, complete sequence
gi|116070110|gb|CY017074.1|[116070110]

35: CY017073
Influenza A virus (A/duck/Viet Nam/18/2005(H5N1)) segment 2, complete sequence
gi|116070107|gb|CY017073.1|[116070107]

36: CY017072
Influenza A virus (A/duck/Viet Nam/18/2005(H5N1)) segment 3, complete sequence
gi|116070105|gb|CY017072.1|[116070105]

37: CY017071
Influenza A virus (A/duck/Viet Nam/18/2005(H5N1)) segment 8, complete sequence
gi|116070102|gb|CY017071.1|[116070102]

38: CY017070
Influenza A virus (A/duck/Viet Nam/18/2005(H5N1)) segment 5, complete sequence
gi|116070100|gb|CY017070.1|[116070100]

39: CY017069
Influenza A virus (A/duck/Viet Nam/18/2005(H5N1)) segment 6, complete sequence
gi|116070098|gb|CY017069.1|[116070098]

40: CY017068
Influenza A virus (A/duck/Viet Nam/18/2005(H5N1)) segment 7, complete sequence
gi|116070095|gb|CY017068.1|[116070095]

41: CY017067
Influenza A virus (A/duck/Viet Nam/18/2005(H5N1)) segment 4, complete sequence
gi|116070093|gb|CY017067.1|[116070093]

42: CY017050
Influenza A virus (A/swan/Slovenia/760/2006(H5N1)) segment 1, complete sequence
gi|116070091|gb|CY017050.1|[116070091]

43: CY017049
Influenza A virus (A/swan/Slovenia/760/2006(H5N1)) segment 2, complete sequence
gi|116070088|gb|CY017049.1|[116070088]

44: CY017048
Influenza A virus (A/swan/Slovenia/760/2006(H5N1)) segment 3, complete sequence
gi|116070086|gb|CY017048.1|[116070086]

45: CY017047
Influenza A virus (A/swan/Slovenia/760/2006(H5N1)) segment 8, complete sequence
gi|116070083|gb|CY017047.1|[116070083]

46: CY017046
Influenza A virus (A/swan/Slovenia/760/2006(H5N1)) segment 5, complete sequence
gi|116070081|gb|CY017046.1|[116070081]

47: CY017045
Influenza A virus (A/swan/Slovenia/760/2006(H5N1)) segment 6, complete sequence
gi|116070079|gb|CY017045.1|[116070079]

48: CY017044
Influenza A virus (A/swan/Slovenia/760/2006(H5N1)) segment 7, complete sequence
gi|116070076|gb|CY017044.1|[116070076]

49: CY017043
Influenza A virus (A/swan/Slovenia/760/2006(H5N1)) segment 4, complete sequence

gi|116070074|gb|CY017043.1|[116070074]

50: CY017042
Influenza A virus (A/cygnus olor/Italy/742/2006(H5N1)) segment 1, complete sequence
gi|116070072|gb|CY017042.1|[116070072]

51: CY017041
Influenza A virus (A/cygnus olor/Italy/742/2006(H5N1)) segment 2, complete sequence
gi|116070069|gb|CY017041.1|[116070069]

52: CY017040
Influenza A virus (A/cygnus olor/Italy/742/2006(H5N1)) segment 3, complete sequence
gi|116070067|gb|CY017040.1|[116070067]

53: CY017039
Influenza A virus (A/cygnus olor/Italy/742/2006(H5N1)) segment 8, complete sequence
gi|116070064|gb|CY017039.1|[116070064]

54: CY017038
Influenza A virus (A/cygnus olor/Italy/742/2006(H5N1)) segment 5, complete sequence
gi|116070062|gb|CY017038.1|[116070062]

55: CY017037
Influenza A virus (A/cygnus olor/Italy/742/2006(H5N1)) segment 6, complete sequence
gi|116070060|gb|CY017037.1|[116070060]

56: CY017036
Influenza A virus (A/cygnus olor/Italy/742/2006(H5N1)) segment 7, complete sequence
gi|116070057|gb|CY017036.1|[116070057]

57: CY017034
Influenza A virus (A/duck/Niger/914/2006(H5N1)) segment 1, complete sequence
gi|116070053|gb|CY017034.1|[116070053]

58: CY017033
Influenza A virus (A/duck/Niger/914/2006(H5N1)) segment 2, complete sequence
gi|116070050|gb|CY017033.1|[116070050]

59: CY017032
Influenza A virus (A/duck/Niger/914/2006(H5N1)) segment 3, complete sequence
gi|116070048|gb|CY017032.1|[116070048]

60: CY017031
Influenza A virus (A/duck/Niger/914/2006(H5N1)) segment 8, complete sequence
gi|116070045|gb|CY017031.1|[116070045]

61: CY017030
Influenza A virus (A/duck/Niger/914/2006(H5N1)) segment 5, complete sequence
gi|116070043|gb|CY017030.1|[116070043]

62: CY017029
Influenza A virus (A/duck/Niger/914/2006(H5N1)) segment 6, complete sequence
gi|116070041|gb|CY017029.1|[116070041]

63: CY017028
Influenza A virus (A/duck/Niger/914/2006(H5N1)) segment 7, complete sequence
gi|116070038|gb|CY017028.1|[116070038]

64: CY017027
Influenza A virus (A/duck/Niger/914/2006(H5N1)) segment 4, complete sequence
gi|116070036|gb|CY017027.1|[116070036]

65: CY016874
Influenza A virus (A/chicken/Viet Nam/10/2005(H5N1)) segment 1, complete sequence
gi|115953527|gb|CY016874.1|[115953527]

66: CY016873
Influenza A virus (A/chicken/Viet Nam/10/2005(H5N1)) segment 2, complete sequence
gi|115953523|gb|CY016873.1|[115953523]

67: CY016872
Influenza A virus (A/chicken/Viet Nam/10/2005(H5N1)) segment 3, complete sequence
gi|115953520|gb|CY016872.1|[115953520]

68: CY016871
Influenza A virus (A/chicken/Viet Nam/10/2005(H5N1)) segment 8, complete sequence
gi|115953516|gb|CY016871.1|[115953516]

69: CY016870
Influenza A virus (A/chicken/Viet Nam/10/2005(H5N1)) segment 5, complete sequence
gi|115953513|gb|CY016870.1|[115953513]

70: CY016869
Influenza A virus (A/chicken/Viet Nam/10/2005(H5N1)) segment 6, complete sequence
gi|115953510|gb|CY016869.1|[115953510]

71: CY016868
Influenza A virus (A/chicken/Viet Nam/10/2005(H5N1)) segment 7, complete sequence
gi|115953506|gb|CY016868.1|[115953506]

72: CY016867
Influenza A virus (A/chicken/Viet Nam/10/2005(H5N1)) segment 4, complete sequence
gi|115953503|gb|CY016867.1|[115953503]

73: CY016842
Influenza A virus (A/chicken/Viet Nam/2/2005(H5N1)) segment 1, complete sequence
gi|115953095|gb|CY016842.1|[115953095]

74: CY016841
Influenza A virus (A/chicken/Viet Nam/2/2005(H5N1)) segment 2, complete sequence
gi|115953092|gb|CY016841.1|[115953092]

75: CY016840
Influenza A virus (A/chicken/Viet Nam/2/2005(H5N1)) segment 3, complete sequence
gi|115953090|gb|CY016840.1|[115953090]

76: CY016839
Influenza A virus (A/chicken/Viet Nam/2/2005(H5N1)) segment 8, complete sequence
gi|115953087|gb|CY016839.1|[115953087]

77: CY016838
Influenza A virus (A/chicken/Viet Nam/2/2005(H5N1)) segment 5, complete sequence
gi|115953085|gb|CY016838.1|[115953085]

78: CY016837
Influenza A virus (A/chicken/Viet Nam/2/2005(H5N1)) segment 6, complete sequence
gi|115953083|gb|CY016837.1|[115953083]

79: CY016836
Influenza A virus (A/chicken/Viet Nam/2/2005(H5N1)) segment 7, complete sequence
gi|115953080|gb|CY016836.1|[115953080]

80: CY016835
Influenza A virus (A/chicken/Viet Nam/2/2005(H5N1)) segment 4, complete sequence
gi|115953078|gb|CY016835.1|[115953078]

81: CY016850
Influenza A virus (A/chicken/Viet Nam/6/2005(H5N1)) segment 1, complete sequence
gi|115953015|gb|CY016850.1|[115953015]

82: CY016849
Influenza A virus (A/chicken/Viet Nam/6/2005(H5N1)) segment 2, complete sequence
gi|115953012|gb|CY016849.1|[115953012]

83: CY016848
Influenza A virus (A/chicken/Viet Nam/6/2005(H5N1)) segment 3, complete sequence
gi|115953010|gb|CY016848.1|[115953010]

84: CY016847
Influenza A virus (A/chicken/Viet Nam/6/2005(H5N1)) segment 8, complete sequence
gi|115953007|gb|CY016847.1|[115953007]

85: CY016846
Influenza A virus (A/chicken/Viet Nam/6/2005(H5N1)) segment 5, complete sequence
gi|115953005|gb|CY016846.1|[115953005]

86: CY016845
Influenza A virus (A/chicken/Viet Nam/6/2005(H5N1)) segment 6, complete sequence
gi|115953003|gb|CY016845.1|[115953003]

87: CY016844
Influenza A virus (A/chicken/Viet Nam/6/2005(H5N1)) segment 7, complete sequence
gi|115953000|gb|CY016844.1|[115953000]

88: CY016843
Influenza A virus (A/chicken/Viet Nam/6/2005(H5N1)) segment 4, complete sequence
gi|115952998|gb|CY016843.1|[115952998]

89: CY016858
Influenza A virus (A/chicken/Viet Nam/8/2005(H5N1)) segment 1, complete sequence
gi|115952912|gb|CY016858.1|[115952912]

90: CY016857
Influenza A virus (A/chicken/Viet Nam/8/2005(H5N1)) segment 2, complete sequence
gi|115952907|gb|CY016857.1|[115952907]

91: CY016856
Influenza A virus (A/chicken/Viet Nam/8/2005(H5N1)) segment 3, complete sequence
gi|115952904|gb|CY016856.1|[115952904]

92: CY016855
Influenza A virus (A/chicken/Viet Nam/8/2005(H5N1)) segment 8, complete sequence
gi|115952899|gb|CY016855.1|[115952899]

93: CY016854
Influenza A virus (A/chicken/Viet Nam/8/2005(H5N1)) segment 5, complete sequence
gi|115952895|gb|CY016854.1|[115952895]

94: CY016853
Influenza A virus (A/chicken/Viet Nam/8/2005(H5N1)) segment 6, complete sequence
gi|115952892|gb|CY016853.1|[115952892]

95: CY016852
Influenza A virus (A/chicken/Viet Nam/8/2005(H5N1)) segment 7, complete sequence
gi|115952888|gb|CY016852.1|[115952888]

96: CY016851
Influenza A virus (A/chicken/Viet Nam/8/2005(H5N1)) segment 4, complete sequence
gi|115952885|gb|CY016851.1|[115952885]

97: CY016866
Influenza A virus (A/chicken/Viet Nam/9/2005(H5N1)) segment 1, complete sequence
gi|115952810|gb|CY016866.1|[115952810]

98: CY016865
Influenza A virus (A/chicken/Viet Nam/9/2005(H5N1)) segment 2, complete sequence
gi|115952806|gb|CY016865.1|[115952806]

99: CY016864
Influenza A virus (A/chicken/Viet Nam/9/2005(H5N1)) segment 3, complete sequence
gi|115952801|gb|CY016864.1|[115952801]

100: CY016863
Influenza A virus (A/chicken/Viet Nam/9/2005(H5N1)) segment 8, complete sequence
gi|115952798|gb|CY016863.1|[115952798]

101: CY016862
Influenza A virus (A/chicken/Viet Nam/9/2005(H5N1)) segment 5, complete sequence
gi|115952795|gb|CY016862.1|[115952795]

102: CY016861
Influenza A virus (A/chicken/Viet Nam/9/2005(H5N1)) segment 6, complete sequence
gi|115952793|gb|CY016861.1|[115952793]

103: CY016860
Influenza A virus (A/chicken/Viet Nam/9/2005(H5N1)) segment 7, complete sequence
gi|115952789|gb|CY016860.1|[115952789]

104: CY016859
Influenza A virus (A/chicken/Viet Nam/9/2005(H5N1)) segment 4, complete sequence
gi|115952786|gb|CY016859.1|[115952786]

105: CY016882
Influenza A virus (A/chicken/Viet Nam/11/2005(H5N1)) segment 1, complete sequence
gi|115952239|gb|CY016882.1|[115952239]

106: CY016881
Influenza A virus (A/chicken/Viet Nam/11/2005(H5N1)) segment 2, complete sequence
gi|115952236|gb|CY016881.1|[115952236]

107: CY016880
Influenza A virus (A/chicken/Viet Nam/11/2005(H5N1)) segment 3, complete sequence
gi|115952233|gb|CY016880.1|[115952233]

108: CY016879
Influenza A virus (A/chicken/Viet Nam/11/2005(H5N1)) segment 8, complete sequence
gi|115952229|gb|CY016879.1|[115952229]

109: CY016878
Influenza A virus (A/chicken/Viet Nam/11/2005(H5N1)) segment 5, complete sequence
gi|115952226|gb|CY016878.1|[115952226]

110: CY016877
Influenza A virus (A/chicken/Viet Nam/11/2005(H5N1)) segment 6, complete sequence
gi|115952224|gb|CY016877.1|[115952224]

111: CY016876
Influenza A virus (A/chicken/Viet Nam/11/2005(H5N1)) segment 7, complete sequence
gi|115952220|gb|CY016876.1|[115952220]

112: CY016875
Influenza A virus (A/chicken/Viet Nam/11/2005(H5N1)) segment 4, complete sequence
gi|115952217|gb|CY016875.1|[115952217]

113: CY016890
Influenza A virus (A/duck/Viet Nam/12/2005(H5N1)) segment 1, complete sequence
gi|115951830|gb|CY016890.1|[115951830]

114: CY016889

Influenza A virus (A/duck/Viet Nam/12/2005(H5N1)) segment 2, complete sequence
gi|115951826|gb|CY016889.1|[115951826]

115: CY016888
Influenza A virus (A/duck/Viet Nam/12/2005(H5N1)) segment 3, complete sequence
gi|115951824|gb|CY016888.1|[115951824]

116: CY016887
Influenza A virus (A/duck/Viet Nam/12/2005(H5N1)) segment 8, complete sequence
gi|115951820|gb|CY016887.1|[115951820]

117: CY016886
Influenza A virus (A/duck/Viet Nam/12/2005(H5N1)) segment 5, complete sequence
gi|115951818|gb|CY016886.1|[115951818]

118: CY016885
Influenza A virus (A/duck/Viet Nam/12/2005(H5N1)) segment 6, complete sequence
gi|115951815|gb|CY016885.1|[115951815]

119: CY016884
Influenza A virus (A/duck/Viet Nam/12/2005(H5N1)) segment 7, complete sequence
gi|115951812|gb|CY016884.1|[115951812]

120: CY016883
Influenza A virus (A/duck/Viet Nam/12/2005(H5N1)) segment 4, complete sequence
gi|115951804|gb|CY016883.1|[115951804]

121: CY016818
Influenza A virus (A/chicken/Cote d'Ivoire/1787-34/2006(H5N1)) segment 1, complete sequence
gi|115609313|gb|CY016818.1|[115609313]

122: CY016817
Influenza A virus (A/chicken/Cote d'Ivoire/1787-34/2006(H5N1)) segment 2, complete sequence
gi|115609310|gb|CY016817.1|[115609310]

123: CY016816
Influenza A virus (A/chicken/Cote d'Ivoire/1787-34/2006(H5N1)) segment 3, complete sequence
gi|115609308|gb|CY016816.1|[115609308]

124: CY016815
Influenza A virus (A/chicken/Cote d'Ivoire/1787-34/2006(H5N1)) segment 8, complete sequence
gi|115609305|gb|CY016815.1|[115609305]

125: CY016814
Influenza A virus (A/chicken/Cote d'Ivoire/1787-34/2006(H5N1)) segment 5, complete sequence
gi|115609303|gb|CY016814.1|[115609303]

126: CY016813
Influenza A virus (A/chicken/Cote d'Ivoire/1787-34/2006(H5N1)) segment 6, complete sequence
gi|115609301|gb|CY016813.1|[115609301]

127: CY016812
Influenza A virus (A/chicken/Cote d'Ivoire/1787-34/2006(H5N1)) segment 7, complete sequence
gi|115609298|gb|CY016812.1|[115609298]

128: CY016811
Influenza A virus (A/chicken/Cote d'Ivoire/1787-34/2006(H5N1)) segment 4, complete sequence
gi|115609292|gb|CY016811.1|[115609292]

129: CY016898
Influenza A virus (A/duck/Viet Nam/20/2005(H5N1)) segment 1, complete sequence
gi|115609777|gb|CY016898.1|[115609777]

130: CY016897
Influenza A virus (A/duck/Viet Nam/20/2005(H5N1)) segment 2, complete sequence

gi|115609774|gb|CY016897.1|[115609774]

131: CY016896
Influenza A virus (A/duck/Viet Nam/20/2005(H5N1)) segment 3, complete sequence
gi|115609771|gb|CY016896.1|[115609771]

132: CY016895
Influenza A virus (A/duck/Viet Nam/20/2005(H5N1)) segment 8, complete sequence
gi|115609768|gb|CY016895.1|[115609768]

133: CY016894
Influenza A virus (A/duck/Viet Nam/20/2005(H5N1)) segment 5, complete sequence
gi|115609765|gb|CY016894.1|[115609765]

134: CY016893
Influenza A virus (A/duck/Viet Nam/20/2005(H5N1)) segment 6, complete sequence
gi|115609762|gb|CY016893.1|[115609762]

135: CY016892
Influenza A virus (A/duck/Viet Nam/20/2005(H5N1)) segment 7, complete sequence
gi|115609759|gb|CY016892.1|[115609759]

136: CY016891
Influenza A virus (A/duck/Viet Nam/20/2005(H5N1)) segment 4, complete sequence
gi|115609756|gb|CY016891.1|[115609756]

137: CY016834
Influenza A virus (A/duck/Viet Nam/1/2005(H5N1)) segment 1, complete sequence
gi|115609715|gb|CY016834.1|[115609715]

138: CY016833
Influenza A virus (A/duck/Viet Nam/1/2005(H5N1)) segment 2, complete sequence
gi|115609712|gb|CY016833.1|[115609712]

139: CY016832
Influenza A virus (A/duck/Viet Nam/1/2005(H5N1)) segment 3, complete sequence
gi|115609710|gb|CY016832.1|[115609710]

140: CY016831
Influenza A virus (A/duck/Viet Nam/1/2005(H5N1)) segment 8, complete sequence
gi|115609707|gb|CY016831.1|[115609707]

141: CY016830
Influenza A virus (A/duck/Viet Nam/1/2005(H5N1)) segment 5, complete sequence
gi|115609704|gb|CY016830.1|[115609704]

142: CY016829
Influenza A virus (A/duck/Viet Nam/1/2005(H5N1)) segment 6, complete sequence
gi|115609702|gb|CY016829.1|[115609702]

143: CY016828
Influenza A virus (A/duck/Viet Nam/1/2005(H5N1)) segment 7, complete sequence
gi|115609699|gb|CY016828.1|[115609699]

144: CY016827
Influenza A virus (A/duck/Viet Nam/1/2005(H5N1)) segment 4, complete sequence
gi|115609697|gb|CY016827.1|[115609697]

145: CY016810
Influenza A virus (A/duck/Cote d'Ivoire/1787-18/2006(H5N1)) segment 1, complete sequence
gi|115609556|gb|CY016810.1|[115609556]

146: CY016809
Influenza A virus (A/duck/Cote d'Ivoire/1787-18/2006(H5N1)) segment 2, complete sequence
gi|115609551|gb|CY016809.1|[115609551]

147: CY016808
Influenza A virus (A/duck/Cote d'Ivoire/1787-18/2006(H5N1)) segment 3, complete sequence
gi|115609549|gb|CY016808.1|[115609549]

148: CY016807
Influenza A virus (A/duck/Cote d'Ivoire/1787-18/2006(H5N1)) segment 8, complete sequence
gi|115609546|gb|CY016807.1|[115609546]

149: CY016806
Influenza A virus (A/duck/Cote d'Ivoire/1787-18/2006(H5N1)) segment 5, complete sequence
gi|115609544|gb|CY016806.1|[115609544]

150: CY016805
Influenza A virus (A/duck/Cote d'Ivoire/1787-18/2006(H5N1)) segment 6, complete sequence
gi|115609542|gb|CY016805.1|[115609542]

151: CY016804
Influenza A virus (A/duck/Cote d'Ivoire/1787-18/2006(H5N1)) segment 7, complete sequence
gi|115609538|gb|CY016804.1|[115609538]

152: CY016803
Influenza A virus (A/duck/Cote d'Ivoire/1787-18/2006(H5N1)) segment 4, complete sequence
gi|115609536|gb|CY016803.1|[115609536]

153: CY016954
Influenza A virus (A/chicken/Nigeria/1047-34/2006(H5N1)) segment 1, complete sequence
gi|115608218|gb|CY016954.1|[115608218]

154: CY016953
Influenza A virus (A/chicken/Nigeria/1047-34/2006(H5N1)) segment 2, complete sequence
gi|115608215|gb|CY016953.1|[115608215]

155: CY016952
Influenza A virus (A/chicken/Nigeria/1047-34/2006(H5N1)) segment 3, complete sequence
gi|115608213|gb|CY016952.1|[115608213]

156: CY016951
Influenza A virus (A/chicken/Nigeria/1047-34/2006(H5N1)) segment 8, complete sequence
gi|115608210|gb|CY016951.1|[115608210]

157: CY016950
Influenza A virus (A/chicken/Nigeria/1047-34/2006(H5N1)) segment 5, complete sequence
gi|115608208|gb|CY016950.1|[115608208]

158: CY016949
Influenza A virus (A/chicken/Nigeria/1047-34/2006(H5N1)) segment 6, complete sequence
gi|115608206|gb|CY016949.1|[115608206]

159: CY016948
Influenza A virus (A/chicken/Nigeria/1047-34/2006(H5N1)) segment 7, complete sequence
gi|115608203|gb|CY016948.1|[115608203]

160: CY016947
Influenza A virus (A/chicken/Nigeria/1047-34/2006(H5N1)) segment 4, complete sequence
gi|115608201|gb|CY016947.1|[115608201]

161: CY016946
Influenza A virus (A/chicken/Nigeria/1047-30/2006(H5N1)) segment 1, complete sequence
gi|115608199|gb|CY016946.1|[115608199]

162: CY016945
Influenza A virus (A/chicken/Nigeria/1047-30/2006(H5N1)) segment 2, complete sequence
gi|115608196|gb|CY016945.1|[115608196]

163: CY016944
Influenza A virus (A/chicken/Nigeria/1047-30/2006(H5N1)) segment 3, complete sequence
gi|115608194|gb|CY016944.1|[115608194]

164: CY016943
Influenza A virus (A/chicken/Nigeria/1047-30/2006(H5N1)) segment 8, complete sequence
gi|115608191|gb|CY016943.1|[115608191]

165: CY016942
Influenza A virus (A/chicken/Nigeria/1047-30/2006(H5N1)) segment 5, complete sequence
gi|115608189|gb|CY016942.1|[115608189]

166: CY016941
Influenza A virus (A/chicken/Nigeria/1047-30/2006(H5N1)) segment 6, complete sequence
gi|115608187|gb|CY016941.1|[115608187]

167: CY016940
Influenza A virus (A/chicken/Nigeria/1047-30/2006(H5N1)) segment 7, complete sequence
gi|115608184|gb|CY016940.1|[115608184]

168: CY016939
Influenza A virus (A/chicken/Nigeria/1047-30/2006(H5N1)) segment 4, complete sequence
gi|115608182|gb|CY016939.1|[115608182]

169: CY016938
Influenza A virus (A/chicken/Nigeria/1047-62/2006(H5N1)) segment 1, complete sequence
gi|115608180|gb|CY016938.1|[115608180]

170: CY016937
Influenza A virus (A/chicken/Nigeria/1047-62/2006(H5N1)) segment 2, complete sequence
gi|115608177|gb|CY016937.1|[115608177]

171: CY016936
Influenza A virus (A/chicken/Nigeria/1047-62/2006(H5N1)) segment 3, complete sequence
gi|115608175|gb|CY016936.1|[115608175]

172: CY016935
Influenza A virus (A/chicken/Nigeria/1047-62/2006(H5N1)) segment 8, complete sequence
gi|115608172|gb|CY016935.1|[115608172]

173: CY016934
Influenza A virus (A/chicken/Nigeria/1047-62/2006(H5N1)) segment 5, complete sequence
gi|115608170|gb|CY016934.1|[115608170]

174: CY016933
Influenza A virus (A/chicken/Nigeria/1047-62/2006(H5N1)) segment 6, complete sequence
gi|115608168|gb|CY016933.1|[115608168]

175: CY016932
Influenza A virus (A/chicken/Nigeria/1047-62/2006(H5N1)) segment 7, complete sequence
gi|115608165|gb|CY016932.1|[115608165]

176: CY016931
Influenza A virus (A/chicken/Nigeria/1047-62/2006(H5N1)) segment 4, complete sequence
gi|115608163|gb|CY016931.1|[115608163]

177: CY016930
Influenza A virus (A/chicken/Nigeria/1047-54/2006(H5N1)) segment 1, complete sequence
gi|115608161|gb|CY016930.1|[115608161]

178: CY016929
Influenza A virus (A/chicken/Nigeria/1047-54/2006(H5N1)) segment 2, complete sequence
gi|115608158|gb|CY016929.1|[115608158]

179: CY016928
Influenza A virus (A/chicken/Nigeria/1047-54/2006(H5N1)) segment 3, complete sequence
gi|115608156|gb|CY016928.1|[115608156]

180: CY016927
Influenza A virus (A/chicken/Nigeria/1047-54/2006(H5N1)) segment 8, complete sequence
gi|115608153|gb|CY016927.1|[115608153]

181: CY016926
Influenza A virus (A/chicken/Nigeria/1047-54/2006(H5N1)) segment 5, complete sequence
gi|115608151|gb|CY016926.1|[115608151]

182: CY016925
Influenza A virus (A/chicken/Nigeria/1047-54/2006(H5N1)) segment 6, complete sequence
gi|115608149|gb|CY016925.1|[115608149]

183: CY016924
Influenza A virus (A/chicken/Nigeria/1047-54/2006(H5N1)) segment 7, complete sequence
gi|115608146|gb|CY016924.1|[115608146]

184: CY016923
Influenza A virus (A/chicken/Nigeria/1047-54/2006(H5N1)) segment 4, complete sequence
gi|115608144|gb|CY016923.1|[115608144]

185: CY016922
Influenza A virus (A/ostrich/Nigeria/1047-25/2006(H5N1)) segment 1, complete sequence
gi|115608142|gb|CY016922.1|[115608142]

186: CY016921
Influenza A virus (A/ostrich/Nigeria/1047-25/2006(H5N1)) segment 2, complete sequence
gi|115608139|gb|CY016921.1|[115608139]

187: CY016920
Influenza A virus (A/ostrich/Nigeria/1047-25/2006(H5N1)) segment 3, complete sequence
gi|115608137|gb|CY016920.1|[115608137]

188: CY016919
Influenza A virus (A/ostrich/Nigeria/1047-25/2006(H5N1)) segment 8, complete sequence
gi|115608134|gb|CY016919.1|[115608134]

189: CY016918
Influenza A virus (A/ostrich/Nigeria/1047-25/2006(H5N1)) segment 5, complete sequence
gi|115608132|gb|CY016918.1|[115608132]

190: CY016917
Influenza A virus (A/ostrich/Nigeria/1047-25/2006(H5N1)) segment 6, complete sequence
gi|115608130|gb|CY016917.1|[115608130]

191: CY016916
Influenza A virus (A/ostrich/Nigeria/1047-25/2006(H5N1)) segment 7, complete sequence
gi|115608127|gb|CY016916.1|[115608127]

192: CY016915
Influenza A virus (A/ostrich/Nigeria/1047-25/2006(H5N1)) segment 4, complete sequence
gi|115608125|gb|CY016915.1|[115608125]

193: CY016914
Influenza A virus (A/chicken/Nigeria/1047-8/2006(H5N1)) segment 1, complete sequence
gi|115608123|gb|CY016914.1|[115608123]

194: CY016913
Influenza A virus (A/chicken/Nigeria/1047-8/2006(H5N1)) segment 2, complete sequence
gi|115608120|gb|CY016913.1|[115608120]

195: CY016912

Influenza A virus (A/chicken/Nigeria/1047-8/2006(H5N1)) segment 3, complete sequence
gi|115608118|gb|CY016912.1|[115608118]

196: CY016911
Influenza A virus (A/chicken/Nigeria/1047-8/2006(H5N1)) segment 8, complete sequence
gi|115608115|gb|CY016911.1|[115608115]

197: CY016910
Influenza A virus (A/chicken/Nigeria/1047-8/2006(H5N1)) segment 5, complete sequence
gi|115608113|gb|CY016910.1|[115608113]

198: CY016909
Influenza A virus (A/chicken/Nigeria/1047-8/2006(H5N1)) segment 6, complete sequence
gi|115608111|gb|CY016909.1|[115608111]

199: CY016908
Influenza A virus (A/chicken/Nigeria/1047-8/2006(H5N1)) segment 7, complete sequence
gi|115608108|gb|CY016908.1|[115608108]

200: CY016907
Influenza A virus (A/chicken/Nigeria/1047-8/2006(H5N1)) segment 4, complete sequence
gi|115608106|gb|CY016907.1|[115608106]

201: CY016906
Influenza A virus (A/duck/Egypt/2253-3/2006(H5N1)) segment 1, complete sequence
gi|115608104|gb|CY016906.1|[115608104]

202: CY016905
Influenza A virus (A/duck/Egypt/2253-3/2006(H5N1)) segment 2, complete sequence
gi|115608101|gb|CY016905.1|[115608101]

203: CY016904
Influenza A virus (A/duck/Egypt/2253-3/2006(H5N1)) segment 3, complete sequence
gi|115608099|gb|CY016904.1|[115608099]

204: CY016903
Influenza A virus (A/duck/Egypt/2253-3/2006(H5N1)) segment 8, complete sequence
gi|115608096|gb|CY016903.1|[115608096]

205: CY016902
Influenza A virus (A/duck/Egypt/2253-3/2006(H5N1)) segment 5, complete sequence
gi|115608094|gb|CY016902.1|[115608094]

206: CY016901
Influenza A virus (A/duck/Egypt/2253-3/2006(H5N1)) segment 6, complete sequence
gi|115608092|gb|CY016901.1|[115608092]

207: CY016900
Influenza A virus (A/duck/Egypt/2253-3/2006(H5N1)) segment 7, complete sequence
gi|115608089|gb|CY016900.1|[115608089]

208: CY016899
Influenza A virus (A/duck/Egypt/2253-3/2006(H5N1)) segment 4, complete sequence
gi|115608087|gb|CY016899.1|[115608087]

209: CY016826
Influenza A virus (A/cygnus olor/Croatia/1/2005(H5N1)) segment 1, complete sequence
gi|115608085|gb|CY016826.1|[115608085]

210: CY016825
Influenza A virus (A/cygnus olor/Croatia/1/2005(H5N1)) segment 2, complete sequence
gi|115608082|gb|CY016825.1|[115608082]

211: CY016824
Influenza A virus (A/cygnus olor/Croatia/1/2005(H5N1)) segment 3, complete sequence

gi|115608080|gb|CY016824.1|[115608080]

212: CY016823
Influenza A virus (A/cygnus olor/Croatia/1/2005(H5N1)) segment 8, complete sequence
gi|115608077|gb|CY016823.1|[115608077]

213: CY016822
Influenza A virus (A/cygnus olor/Croatia/1/2005(H5N1)) segment 5, complete sequence
gi|115608075|gb|CY016822.1|[115608075]

214: CY016821
Influenza A virus (A/cygnus olor/Croatia/1/2005(H5N1)) segment 6, complete sequence
gi|115608073|gb|CY016821.1|[115608073]

215: CY016820
Influenza A virus (A/cygnus olor/Croatia/1/2005(H5N1)) segment 7, complete sequence
gi|115608070|gb|CY016820.1|[115608070]

216: CY016819
Influenza A virus (A/cygnus olor/Croatia/1/2005(H5N1)) segment 4, complete sequence
gi|115608068|gb|CY016819.1|[115608068]

217: CY016802
Influenza A virus (A/mallard/Italy/835/2006(H5N1)) segment 1, complete sequence
gi|115608066|gb|CY016802.1|[115608066]

218: CY016801
Influenza A virus (A/mallard/Italy/835/2006(H5N1)) segment 2, complete sequence
gi|115608063|gb|CY016801.1|[115608063]

219: CY016800
Influenza A virus (A/mallard/Italy/835/2006(H5N1)) segment 3, complete sequence
gi|115608061|gb|CY016800.1|[115608061]

220: CY016799
Influenza A virus (A/mallard/Italy/835/2006(H5N1)) segment 8, complete sequence
gi|115608058|gb|CY016799.1|[115608058]

221: CY016798
Influenza A virus (A/mallard/Italy/835/2006(H5N1)) segment 5, complete sequence
gi|115608056|gb|CY016798.1|[115608056]

222: CY016797
Influenza A virus (A/mallard/Italy/835/2006(H5N1)) segment 6, complete sequence
gi|115608054|gb|CY016797.1|[115608054]

223: CY016796
Influenza A virus (A/mallard/Italy/835/2006(H5N1)) segment 7, complete sequence
gi|115608051|gb|CY016796.1|[115608051]

224: CY016795
Influenza A virus (A/mallard/Italy/835/2006(H5N1)) segment 4, complete sequence
gi|115608049|gb|CY016795.1|[115608049]

225: CY016794
Influenza A virus (A/chicken/Afghanistan/1207/2006(H5N1)) segment 1, complete sequence
gi|115608047|gb|CY016794.1|[115608047]

226: CY016793
Influenza A virus (A/chicken/Afghanistan/1207/2006(H5N1)) segment 2, complete sequence
gi|115608044|gb|CY016793.1|[115608044]

227: CY016792
Influenza A virus (A/chicken/Afghanistan/1207/2006(H5N1)) segment 3, complete sequence
gi|115608042|gb|CY016792.1|[115608042]

228: CY016791
Influenza A virus (A/chicken/Afghanistan/1207/2006(H5N1)) segment 8, complete sequence
gi|115608039|gb|CY016791.1|[115608039]

229: CY016790
Influenza A virus (A/chicken/Afghanistan/1207/2006(H5N1)) segment 5, complete sequence
gi|115608037|gb|CY016790.1|[115608037]

230: CY016789
Influenza A virus (A/chicken/Afghanistan/1207/2006(H5N1)) segment 6, complete sequence
gi|115608035|gb|CY016789.1|[115608035]

231: CY016788
Influenza A virus (A/chicken/Afghanistan/1207/2006(H5N1)) segment 7, complete sequence
gi|115608032|gb|CY016788.1|[115608032]

232: CY016787
Influenza A virus (A/chicken/Afghanistan/1207/2006(H5N1)) segment 4, complete sequence
gi|115608030|gb|CY016787.1|[115608030]

233: CY016786
Influenza A virus (A/cygnus cygnus/Iran/754/2006(H5N1)) segment 1, complete sequence
gi|115608028|gb|CY016786.1|[115608028]

234: CY016785
Influenza A virus (A/cygnus cygnus/Iran/754/2006(H5N1)) segment 2, complete sequence
gi|115608025|gb|CY016785.1|[115608025]

235: CY016784
Influenza A virus (A/cygnus cygnus/Iran/754/2006(H5N1)) segment 3, complete sequence
gi|115608023|gb|CY016784.1|[115608023]

236: CY016783
Influenza A virus (A/cygnus cygnus/Iran/754/2006(H5N1)) segment 8, complete sequence
gi|115608020|gb|CY016783.1|[115608020]

237: CY016782
Influenza A virus (A/cygnus cygnus/Iran/754/2006(H5N1)) segment 5, complete sequence
gi|115608018|gb|CY016782.1|[115608018]

238: CY016781
Influenza A virus (A/cygnus cygnus/Iran/754/2006(H5N1)) segment 6, complete sequence
gi|115608016|gb|CY016781.1|[115608016]

239: CY016780
Influenza A virus (A/cygnus cygnus/Iran/754/2006(H5N1)) segment 7, complete sequence
gi|115608013|gb|CY016780.1|[115608013]

240: CY016779
Influenza A virus (A/cygnus cygnus/Iran/754/2006(H5N1)) segment 4, complete sequence
gi|115608011|gb|CY016779.1|[115608011]

241: CY016307
Influenza A virus (A/chicken/Sudan/1784-10/2006(H5N1)) segment 1, complete sequence
gi|115344810|gb|CY016307.1|[115344810]

242: CY016306
Influenza A virus (A/chicken/Sudan/1784-10/2006(H5N1)) segment 2, complete sequence
gi|115344807|gb|CY016306.1|[115344807]

243: CY016305
Influenza A virus (A/chicken/Sudan/1784-10/2006(H5N1)) segment 3, complete sequence
gi|115344805|gb|CY016305.1|[115344805]

244: CY016304
Influenza A virus (A/chicken/Sudan/1784-10/2006(H5N1)) segment 8, complete sequence
gi|115344802|gb|CY016304.1|[115344802]

245: CY016303
Influenza A virus (A/chicken/Sudan/1784-10/2006(H5N1)) segment 5, complete sequence
gi|115344800|gb|CY016303.1|[115344800]

246: CY016302
Influenza A virus (A/chicken/Sudan/1784-10/2006(H5N1)) segment 6, complete sequence
gi|115344798|gb|CY016302.1|[115344798]

247: CY016301
Influenza A virus (A/chicken/Sudan/1784-10/2006(H5N1)) segment 7, complete sequence
gi|115344795|gb|CY016301.1|[115344795]

248: CY016300
Influenza A virus (A/chicken/Sudan/1784-10/2006(H5N1)) segment 4, complete sequence
gi|115344793|gb|CY016300.1|[115344793]

249: CY016299
Influenza A virus (A/chicken/Sudan/1784-7/2006(H5N1)) segment 1, complete sequence
gi|115344791|gb|CY016299.1|[115344791]

250: CY016298
Influenza A virus (A/chicken/Sudan/1784-7/2006(H5N1)) segment 2, complete sequence
gi|115344788|gb|CY016298.1|[115344788]

251: CY016297
Influenza A virus (A/chicken/Sudan/1784-7/2006(H5N1)) segment 3, complete sequence
gi|115344786|gb|CY016297.1|[115344786]

252: CY016296
Influenza A virus (A/chicken/Sudan/1784-7/2006(H5N1)) segment 8, complete sequence
gi|115344783|gb|CY016296.1|[115344783]

253: CY016295
Influenza A virus (A/chicken/Sudan/1784-7/2006(H5N1)) segment 5, complete sequence
gi|115344781|gb|CY016295.1|[115344781]

254: CY016294
Influenza A virus (A/chicken/Sudan/1784-7/2006(H5N1)) segment 6, complete sequence
gi|115344779|gb|CY016294.1|[115344779]

255: CY016293
Influenza A virus (A/chicken/Sudan/1784-7/2006(H5N1)) segment 7, complete sequence
gi|115344776|gb|CY016293.1|[115344776]

256: CY016292
Influenza A virus (A/chicken/Sudan/1784-7/2006(H5N1)) segment 4, complete sequence
gi|115344774|gb|CY016292.1|[115344774]

257: CY016291
Influenza A virus (A/chicken/Nigeria/957-20/2006(H5N1)) segment 1, complete sequence
gi|115344772|gb|CY016291.1|[115344772]

258: CY016290
Influenza A virus (A/chicken/Nigeria/957-20/2006(H5N1)) segment 2, complete sequence
gi|115344769|gb|CY016290.1|[115344769]

259: CY016289
Influenza A virus (A/chicken/Nigeria/957-20/2006(H5N1)) segment 3, complete sequence
gi|115344767|gb|CY016289.1|[115344767]

260: CY016288
Influenza A virus (A/chicken/Nigeria/957-20/2006(H5N1)) segment 8, complete sequence
gi|115344764|gb|CY016288.1|[115344764]

261: CY016287
Influenza A virus (A/chicken/Nigeria/957-20/2006(H5N1)) segment 5, complete sequence
gi|115344762|gb|CY016287.1|[115344762]

262: CY016286
Influenza A virus (A/chicken/Nigeria/957-20/2006(H5N1)) segment 6, complete sequence
gi|115344760|gb|CY016286.1|[115344760]

263: CY016285
Influenza A virus (A/chicken/Nigeria/957-20/2006(H5N1)) segment 7, complete sequence
gi|115344757|gb|CY016285.1|[115344757]

264: CY016284
Influenza A virus (A/chicken/Nigeria/957-20/2006(H5N1)) segment 4, complete sequence
gi|115344755|gb|CY016284.1|[115344755]

265: CY016283
Influenza A virus (A/chicken/Nigeria/641/2006(H5N1)) segment 1, complete sequence
gi|115344753|gb|CY016283.1|[115344753]

266: CY016282
Influenza A virus (A/chicken/Nigeria/641/2006(H5N1)) segment 2, complete sequence
gi|115344750|gb|CY016282.1|[115344750]

267: CY016281
Influenza A virus (A/chicken/Nigeria/641/2006(H5N1)) segment 3, complete sequence
gi|115344748|gb|CY016281.1|[115344748]

268: CY016280
Influenza A virus (A/chicken/Nigeria/641/2006(H5N1)) segment 8, complete sequence
gi|115344745|gb|CY016280.1|[115344745]

269: CY016279
Influenza A virus (A/chicken/Nigeria/641/2006(H5N1)) segment 5, complete sequence
gi|115344743|gb|CY016279.1|[115344743]

270: CY016278
Influenza A virus (A/chicken/Nigeria/641/2006(H5N1)) segment 6, complete sequence
gi|115344741|gb|CY016278.1|[115344741]

271: CY016277
Influenza A virus (A/chicken/Nigeria/641/2006(H5N1)) segment 7, complete sequence
gi|115344738|gb|CY016277.1|[115344738]

272: CY016276
Influenza A virus (A/chicken/Nigeria/641/2006(H5N1)) segment 4, complete sequence
gi|115344736|gb|CY016276.1|[115344736]
